# Beyond Equal-Power Sparse NOMA: Two User Classes and Closed-Form Bounds on the Achievable Region †

**DOI:** 10.3390/e24020227

**Published:** 2022-01-31

**Authors:** Benjamin M. Zaidel, Ori Shental, Shlomo Shamai (Shitz)

**Affiliations:** 1 The Alexander Kofkin Faculty of Engineering, Bar-Ilan University, Ramat-Gan 5290002, Israel; 2 Qualcomm Inc., 5775 Morehouse Drive, San Diego, CA 92121, USA; oshental@qti.qualcomm.com; 3 Department of Electrical and Computer Engineering, Technion—Israel Institute of Technology, Haifa 3200003, Israel; sshlomo@ee.technion.ac.il

**Keywords:** non-orthogonal multiple-access, entropy power inequality, sparse code-domain NOMA

## Abstract

Non-orthogonal multiple access (NOMA) is a promising technology for future beyond-5G wireless networks, whose fundamental information-theoretic limits are yet to be fully explored. Considering regular sparse code-domain NOMA (with a fixed and finite number of orthogonal resources allocated to any designated user and vice versa), this paper extends previous results by the authors to a setting comprising two classes of users with different power constraints. Explicit rigorous *closed-form* analytical inner and outer bounds on the achievable rate (total class throughput) region in the large-system limit are derived and comparatively investigated in extreme-SNR regimes. The inner bound is based on the conditional vector entropy power inequality (EPI), while the outer bound relies on a recent strengthened version of the EPI. Valuable insights are provided into the potential performance gains of regular sparse NOMA in practically oriented settings, comprising, e.g., a combination of low-complexity devices and broadband users with higher transmit power capabilities, or combinations of cell-edge and cell-center users. The conditions for superior performance over dense code-domain NOMA (taking the form of randomly spread code-division multiple access), as well as a relatively small gap to the ultimate performance limits, are identified. The proposed bounds are also applicable for the analysis of interference networks, e.g., Wyner-type cellular models.

## 1. Introduction

Non-orthogonal multiple access (NOMA) is a key enabler in the design of future overloaded beyond-5G communication systems with many more designated users than available physical resources, precluding the conventional orthogonal multiple access (OMA) paradigm [1,2,3,4] (see also [5] for a very recent technology review). The main potential appeal of NOMA over OMA stems from either supporting more simultaneous users or, in lieu, facilitating higher user throughputs when orthogonality is practically unsustainable. NOMA technologies generally comprise two main manifestations, power-domain NOMA and code-domain NOMA. Power-domain NOMA essentially relies on direct superposition of the transmitted signals, successive interference cancellation (SIC) at the receivers and appropriate power allocation to different users in order to achieve desired performance objectives [1,2,6,7]. Under the code-domain NOMA paradigm, the users’ signals are distinguished by different spreading signatures chosen to facilitate efficient multiuser detection (MUD) at the receivers (see, e.g., [2,8]). In particular, sparse NOMA, or low-density code-domain (LDCD) NOMA, has gained considerable interest in recent years due to its appealing attributes. Relying on sparse spreading signatures, sparse NOMA potentially facilitates enhanced spectral efficiency with practical receiver implementation based on sparsity exploiting iterative message passing algorithms (MPAs), similarly to the ones empowering the efficient decoding of low-density parity-check (LDPC) codes. The interested reader is referred to the insightful surveys [1,2] for details about the utilization of MPAs in NOMA, along with their concrete application for sparse NOMA [9,10,11], including sparse-code multiple access (SCMA) [12]. Different designs of sparse spreading signatures and their impact on MUD error-rate performance are discussed, e.g., in [11,13,14] and references therein.

Transmission schemes *combining* power-domain NOMA and SCMA were also recently proposed, e.g., in [15,16] for the cellular downlink channel and in [17,18,19,20,21] for the uplink channel. Therein, the main objective is to identify efficient centralized algorithms for joint resource and power allocation, that attempt to maximize the SCMA achievable throughput under independent Rayleigh fading and certain simplifying assumptions (viz., independent Gaussian signaling over each utilized physical resource, full synchronization and perfect channel state information). Fairness and quality-of-service constraints may also be incorporated into the optimization algorithm (e.g., [19,20,21]). The performance of the proposed algorithms is then evaluated by means of numerical simulations. More involved network configurations have also been considered recently, e.g., system models encompassing relays (see [22] and references therein for an exhaustive literature survey). Relaying may either appear in the form of dedicated network elements, e.g., [22,23] or, alternatively, by means of user cooperation, e.g., [24,25,26,27]. In this framework, focusing on power-domain NOMA with SIC, the notion of virtual full-duplex (VFD) relaying [28] has gained particular interest as means to circumvent the implementation challenges of true full-duplex operation; see, e.g., [22,25,26,27]. The impact of imperfect SIC and residual inter-relay interference in this framework was also recently considered in [26].

Notwithstanding their great practical promise and potential, sparse NOMA techniques often pose serious analytical challenges and their information-theoretic performance limits are not easily tractable even in the simplest settings. Typically, tools from random matrix theory or statistical physics are harnessed for their analysis [29,30,31,32], while considering the asymptotic large-system limit, where both the number of users and the number of available resources grow large, while retaining a fixed ratio (see, e.g., [33,34,35]). The obtained results typically yield *excellent approximations* for the expected performance with finite (and quite moderate) system dimensions [29,30].

Sparse NOMA is dubbed: *regular* when a *fixed* (and finite) number of orthogonal resources is allocated to any designated user and each resource is used by a fixed number of users; *irregular* when the respective numbers are random and only kept fixed on average [33]. In the literature, one can also find a *partly regular* version of the sparse NOMA setup where each user occupies a fixed number of resources and each resource is used by a random, yet fixed on average, number of users (or vice versa) [34,36].

In a recent line of works by the authors [37,38,39], the particular manifestation of code-domain NOMA known as *regular sparse NOMA* has been investigated and its asymptotic spectral efficiency has been derived in *closed form* [38,39] (see Section 2 for a precise characterization of the underlying asymptotic large-system limit and [38,39] for a discussion on what distinguishes this particular setting from previous analyses). Therein, a generic setup of a (non-fading) Gaussian vector multiple-access channel (MAC) with equal-power users was considered, representing the case of a single-cell uplink model with fully coordinated grant-based access, and the scheme was analytically proven to substantially decrease the gap to the ultimate capacity limit of overloaded systems. Furthermore, regular sparse NOMA was proven by the authors to outperform the dense code-domain NOMA alternative [40,41], along with its irregular and partly regular sparse counterparts [33,34]. Hence, regular sparse NOMA seems to exhibit a rare combination of information-theoretic superiority and *computational feasibility* (dense code-domain NOMA is operationally equivalent to randomly spread code-division multiple access (RS-CDMA) [40,41], for which achieving the optimal spectral efficiency becomes *prohibitively complex* in large systems [42]). However, analyzing the merits of regular sparse NOMA beyond the generic equal-power Gaussian vector MAC setting is still a challenging, yet of utmost importance, open problem, which does not seem to lend itself to closed-form characterization, as in [38,39].

Motivated by this noteworthy challenge, the current paper takes a step further towards a generalization of the fundamental result of [38,39]. To this end, the focus remains on regular sparse NOMA within a single-cell Gaussian uplink (MAC) model, but the single-class information-theoretic analysis of [37,38,39] is extended to a looser, yet more realistic, setting comprising *two* user classes distinguished by their received powers. Again, the large-system limit is considered and our main contribution is the derivation of *closed-form bounds* on the achievable class-rate (total throughput) region, which are both insightful and analytically tractable. An inner bound is first derived based on the standard conditional vector entropy power inequality (EPI) [43]. A derivation of an outer bound follows, while relying on a recent strengthened version of the EPI by Courtade [44,45]. Both bounds are *tight* with respect to the individual achievable class-throughput constraints and only differ in the achievable sum-rate (total throughput) constraint. The key tool in the derivations is a noise-split “trick”, that, when combined with the EPI, induces closed-form bounds expressed in terms of single-class achievable throughputs [38,39]. A simplified outer bound, which does not rely on the EPI, is then presented for load-symmetric settings (under some mild technical assumptions). This bound turns out to be tighter in certain cases. An in-depth elucidative investigation of the corresponding lower and upper bounds on the achievable sum rate in extreme-SNR regimes is also provided, which, by means of appropriate approximations [41], identifies conditions under which the bounds are useful and a superior performance over dense code-domain NOMA is guaranteed. Conditions for attaining a relatively small gap to the ultimate performance limits are also discussed.

Our contribution provides valuable insights into the potential performance gains of regular sparse NOMA in several timely use cases of interest. One particular example represents a 5G-and-beyond scenario, where the two user classes respectively correspond, say, to low-complexity devices with stringent power constraints (e.g., Internet-of-Things applications) and to broadband users with higher transmit power capabilities. Another applicable use case is a single cell with users located at the extremes of either the cell center or the cell edge. Our analysis is also applicable to a compelling combination of power-domain NOMA [2,6] and code-domain NOMA, which has only recently started to attract attention in the literature [15,16,17,18,19,20,21], as discussed above. Accordingly, the corner points of the achievable region bounds correspond to a SIC scheme between the two user classes, while incorporating near-optimal joint iterative decoding (MPA) within each class. In fact, by this interpretation, our analysis provides, in a sense, an *analytical* benchmark for the setting considered in [20] (see also [18]), under the simplifying assumptions of non-fading channels and full symmetry among the users in each user class. Note that, in the *absence* of fading, the corresponding regular SCMA achievable sum rate, while assuming independent Gaussian signaling over each utilized resource, trivially coincides with the Cover–Wyner sum capacity (see, e.g., [43] and Section 3.3). Hence, our analytical bounds quantify the gap from the ultimate performance limit induced by employing regular sparse spreading signatures and may serve as reference for practical schemes that aim to approach the sum-capacity limit using the SCMA paradigm, e.g., [17,18,19,20,21]. Note that, in the presence of fading, characterizing the achievable rate region of the two-user-class system considered in this paper is still a formidable open problem yet to be explored; hence, a direct and explicit comparison with practical achievable sum rates reported in works, such as [20], cannot be performed at this stage. Yet another applicable model is a two-cell interference network, where the two user classes represent, respectively, the local cell users and the users operating in the adjacent interfering cell. This may be further extended, e.g., to Wyner-type cellular models with single-cell processing [46,47,48,49].

This paper is organized as follows: Section 2 describes the underlying system model and the random graph models employed to construct the regular sparse spreading signatures. Section 3 presents a general statement of the class-throughput achievable region and the corresponding closed-form analytical inner and outer bounds. Section 4 is devoted to a comparative extreme-SNR characterization of the lower and upper bounds on the total achievable sum rate. Illustrative numerical results are provided in Section 5. Finally, Section 6 ends this paper with some concluding remarks. Detailed proofs and some technical observations are deferred to the appendices.

## 2. System Model

*Notation:* We use boldface lower-case letters to denote vectors and boldface uppercase letters to denote matrices. ${\left[M\right]}_{ij}$ denotes the (i,j)-th entry of the matrix M. $M^{T}$ denotes the transpose of M, while $M^{†}$ denotes the corresponding conjugate (Hermitian) transpose. ⊗ denotes the Kronecker product. $I_{N}$ denotes the *N*-dimensional identity matrix. CN(μ,Σ) designates the distribution of a proper circularly symmetric complex Gaussian random vector with mean μ and covariance matrix Σ. $\delta_{x}$ designates the probability distribution of a single mass at *x*. Equality in distribution is denoted by $\overset{d}{=}$, stating that the *distribution* of the random variables on both sides of the equality sign is the same. $E\left\{\cdot \right\}$ denotes statistical expectation and $E_{X}\left\{\cdot \right\}$ designates that the expectation is taken with respect to the distribution of the random variable *X*. h(·) denotes differential entropy and I(·;·) denotes mutual information. For any ϵ∈(0,1), we use the notation $\bar{\epsilon }≜1-\epsilon$. Base-2 logarithms are used throughout this paper unless otherwise stated (in which case the base of the logarithm is explicitly designated). For the sake of clarity, we use ln(·) to denote the natural logarithm.

We consider a MAC, representing a single-cell uplink, where the users belong to either of two different classes distinguished by their received powers (henceforth referred to as “Class 1” and “Class 2”). Within each class, all users are assumed to be received at the *same* power level. The users’ signals are multiplexed over *N* shared orthogonal dimensions (resources), which may represent, e.g., orthogonal time–frequency slots. However, it is important to emphasize here that the setting is quite general and applies to any orthogonal coordinate system; therefore, the dimensions are, by no means, restricted to the time–frequency domain. Let $K_{1}$ and $K_{2}$ denote the number of users in Class 1 and Class 2, respectively, and let $\beta_{i}≜K_{i}/N$, i=1,2 denote the respective loads (users per resource). The total number of users is denoted by $K≜K_{1}+K_{2}$ and the total system load reads $\beta ≜K/N=\beta_{1}+\beta_{2}$.

Focusing on a generic non-fading Gaussian channel model, the *N*-dimensional received signal at some arbitrary time instance reads

(1) $$y=\sqrt{\frac{{\mathrm{snr}}_{1}}{d}}A_{1}x_{1}+\sqrt{\frac{{\mathrm{snr}}_{2}}{d}}A_{2}x_{2}+z\;,$$

where $x_{i}$, i=1,2, is a $K_{i}$-dimensional complex vector comprising the coded symbols of the users in Class *i*. Assuming Gaussian signaling, full symmetry, fixed powers and no cooperation among encoders corresponding to different users, the input vector $x_{i}$ is distributed as $x_{i}∼\mathrm{CN}\left(0,I_{K_{i}}\right)$. The matrix $A_{i}$ represents the $N\times K_{i}$ *sparse* signature matrix of Class-*i* users, where the *k*th column represents the spreading signature of user *k* in Class *i*. The non-zero entries of $A_{i}$ designate the corresponding user-resource mapping, namely, user *k* in Class *i* occupies resource *n* if ${\left[A_{i}\right]}_{nk}\neq 0$. Specifically, we adhere to the regular sparse NOMA paradigm [37,38,39], where, for each Class *i*, i=1,2, due to the sparsity of $A_{i}$, only a few of the users’ signals collide over any given orthogonal resource. The regularity assumption generally dictates that each column of $A_{i}$ (respectively, row) has *exactly* $d_{i}\geq 2\in N^{+}$ (respectively, $\beta_{i}d_{i}$) non-zero entries. However, for notational simplicity, we assume henceforth that $d_{1}=d_{2}=d$, while noting that extension of the analysis to the case where $A_{1}$ and $A_{2}$ have a different fixed number of non-zero column entries is straightforward (hence omitted). Therefore, *d* takes the role of the *system’s* sparsity parameter. We assume here that $\beta_{i}$ is chosen so that $\beta_{i}d\geq 2\in N^{+}$, i=1,2, in order to avoid degenerate settings. The non-zero entries of $A_{i}$ are assumed to be independent and identically distributed (i.i.d.), but may otherwise arbitrarily reside on the unit circle in the complex plane, in complete adherence to [38,39]. Thus, the normalization in (1) ensures that the columns of $\frac{1}{\sqrt{d}}A_{i}$ have unit norm. We also assume here that the signature matrices $A_{1}$ and $A_{2}$ are perfectly known at the receiving end and uniformly chosen, respectively, randomly and independently per each channel use and each user class *i*, from the set of $\left(\beta_{i}d,d\right)$-regular matrices. This assumption is only introduced here for the sake of concreteness and the setting, in fact, generalizes verbatim to the case where the signature matrix selection process is stationary and ergodic. Finally, $z∼\mathrm{CN}(0,I_{N})$ denotes the *N*-dimensional circularly symmetric complex additive white-Gaussian-noise (AWGN) vector at the receiving end. Thus, the parameter ${\mathrm{snr}}_{i}$ in (1), i=1,2, designates the received signal-to-noise ratio (SNR) of each of the users in Class *i*.

A key additional underlying assumption, which follows [38,39], is that the signature matrices ${\left\{A_{i}\right\}}_{i=1,2}$ can be associated with the *adjacency matrices* of certain random $\left(\beta_{i}d,d\right)$-semiregular bipartite (factor) graphs ${\left\{A_{i}\right\}}_{i=1,2}$, with special properties to be stated next. To this end, we first introduce the following two definitions.

**Definition** **1** (Locally Tree-Like Graphs [50,51])**.** *Let $G^{⋆}$ denote the space of rooted isomorphism classes of rooted connected graphs. A sequence of random graphs $G_{n}$, n≥1, in the space $G^{⋆}$, with a root vertex $v_{n}$ chosen uniformly at random from the vertex set of $G_{n}$, is said to converge locally (weakly) to a certain random rooted tree (T,o), if, for each r≥0, the sequence of balls $B_{r}^{G_{n}}\left(v_{n}\right)$ with radius r (in graph distance) around $v_{n}$ converges in law to $B_{r}^{T}\left(o\right)$ in the space $G^{⋆}$. A more precise mathematical definition can be found, e.g., in [50,51].*

**Definition** **2** (Bipartite Galton–Watson Tree (BGWT) [50])**.** *A Galton–Watson tree (GWT) with* degree distribution $F_{*}$ is a rooted random tree obtained by a Galton–Watson branching process, where the root has offspring distribution $F_{*}$ and all other genitors have offspring distribution F, where (assuming $\sum_{k}kF_{*}\left(k\right)<\infty$)

(2) $$F\left(k-1\right)=\frac{kF_{*}\left(k\right)}{\sum_{k}kF_{*}\left(k\right)}\;,\;k\geq 1\;.$$

A BGWT with degree distribution $\left(F_{*},G_{*}\right)$ and parameter p is obtained from a Galton–Watson branching process with alternated degree distribution. Namely, with probability p, the root has offspring distribution $F_{*}$, all odd generation genitors have an offspring distribution G (related to $G_{*}$ analogously to F) and all even generation genitors (apart from the root) have an offspring distribution F. Similarly, with probability 1−p, the root has offspring distribution $G_{*}$ and the offspring distributions of all odd and even generation genitors are switched. See [50] for a more elaborate discussion.

We now further assume that the random graphs ${\left\{A_{i}\right\}}_{i=1,2}$ associated with the signature matrices are locally tree-like and converge in the large-system limit to BGWTs having degree distribution $\left(\delta_{\beta_{i}d},\delta_{d}\right)$ and parameter $\frac{1}{1+\beta_{i}}$ as a weak limit, where i=1,2. The term “large-system limit” refers here to the regime where $N,K_{1},K_{2}\to \infty$ while fixing $K_{i}/N=\beta_{i}$, i=1,2. We use henceforth the shorthand notation “N→∞” to designate this limiting regime.

From a practical perspective, it is important to note here that the aforementioned locally tree-like property is valid, e.g., for regular LDPC codes [50,52] and it essentially implies that, for large dimensions, short cycles are rare, which facilitates the use of iterative near-optimal multiuser detection algorithms (while applying MPAs over the underlying factor graphs). Moreover, the sparse signature matrices can in fact be constructed as weighted parity-check matrices of regular LDPC codes, which we, in fact, employ using Gallager’s construction [53] to produce the finite dimensional simulation results in Section 5 (see therein).

## 3. The Achievable Rate Region

### 3.1. Preliminaries

For the sake of completeness, we first review the main result of [38,39] (adapted to the current system model), which constitutes the basis for the analysis in the sequel. Let us consider a *single-class* channel model (cf. ( 1)) as follows:

(3) $$y=\sqrt{\frac{{\mathrm{snr}}_{i}}{d}}A_{i}x_{i}+z\;,\;i=1,2\;.$$

**Theorem** **1** ([38], Theorem 3)**.** *Let $A_{i}$, i=1,2, be a sparse random $N\times K_{i}$ matrix with exactly $2\leq d\in N^{+}<\infty$ (respectively, $2\leq \beta_{i}d\in N^{+}<\infty$) non-zero entries in each column (respectively, row), independent and identically arbitrarily distributed over the unit circle in C. Assume that the $\left(\beta_{i}d,d\right)$-semiregular bipartite graph $A_{i}$ associated with $A_{i}$ is locally tree-like with the limiting properties specified in Section 2. Let $\alpha ≜\frac{d-1}{d}$ and $\gamma_{i}≜\frac{\beta_{i}d-1}{d}$, i=1,2. Further, let ${\tilde{\beta }}_{i}≜\frac{\alpha }{\gamma_{i}}$ and $\zeta_{i}≜\frac{\beta_{i}d}{\gamma_{i}}$, i=1,2. Then, the normalized conditional input–output mutual information*

(4) $$\frac{1}{N}I\left(x_{i};y|A_{i}\right)=\frac{1}{N}E\left\{\log\det\left(I_{N}+\frac{{\mathrm{snr}}_{i}}{d}A_{i}A_{i}^{†}\right)\right\}\;,\;i=1,2,$$

*converges, as N→∞, to*

(5) Ci(snri)≜Copt(snri,βi,d)=(βi−1)log1+αsnri−14F(γisnri,βi˜)Ci==+βi(d−1)+12log1+(γi+α)snri−14F(γisnri,βi˜)=−βi(d−1)−12log(1+βidsnri)2G(γisnri,ζi,β˜i),i=1,2,

*where (cf. [40])*

(6) $$F\left(x,z\right)≜{\left(\sqrt{x{\left(1+\sqrt{z}\right)}^{2}+1}-\sqrt{x{\left(1-\sqrt{z}\right)}^{2}+1}\right)}^{2}\;,$$

*and, for $x,y,z\in R^{+},\;y\geq {\left(1+\sqrt{z}\right)}^{2}$,*

(7) $$G\left(x,y,z\right)≜\;{\left(\frac{\sqrt{\left(y-{\left(1-\sqrt{z}\right)}^{2}\right)\left(x{\left(1+\sqrt{z}\right)}^{2}+1\right)}-\sqrt{\left(y-{\left(1+\sqrt{z}\right)}^{2}\right)\left(x{\left(1-\sqrt{z}\right)}^{2}+1\right)}}{\sqrt{y-{\left(1-\sqrt{z}\right)}^{2}}-\sqrt{y-{\left(1+\sqrt{z}\right)}^{2}}}\right)}^{2}\;.$$

Let $R_{i}$ denote the normalized spectral efficiency (*total* throughput) in bit/sec/Hz of the users in Class *i*, i=1,2. Let R denote the achievable region of rate pairs $\left(R_{1},R_{2}\right)$ for the channel (1). Then, by the standard properties of the MAC capacity region (e.g., [43]), it follows that

(8) $$\begin{array}{cc} R=\{\left(R_{1},R_{2}\right):R_{1} & \leq \frac{1}{N}I\left(x_{1};y|x_{2},{\left\{A_{i}\right\}}_{i=1,2}\right),\\ R_{2} & \leq \frac{1}{N}I\left(x_{2};y|x_{1},{\left\{A_{i}\right\}}_{i=1,2}\right),\\ R_{1}+R_{2} & \leq \frac{1}{N}I\left(x_{1},x_{2};y|{\left\{A_{i}\right\}}_{i=1,2}\right)\}. \end{array}$$

In the large-system limit, the two constraints on the (class) individual rates can be characterized explicitly and in closed form by means of Theorem 1. Namely, the two bounds converge to the following limits:

(9) $$\frac{1}{N}I\left(x_{i};y|x_{j},{\left\{A_{i}\right\}}_{i=1,2}\right)\underset{N\to \infty ;\;i,j=1,2;\;i\neq j}{\to }C_{i}\left({\mathrm{snr}}_{i}\right).\;$$

Note that $C_{i}\left({\mathrm{snr}}_{i}\right)$ specifies, in the large-system limit, the normalized spectral efficiency with optimum processing in bit/sec/Hz of the users in Class *i*, given the signals transmitted by the users in Class *j*, j≠i [38,39]. However, unfortunately, a corresponding limiting result for the maximum achievable sum rate $\frac{1}{N}I\left(x_{1},x_{2};y|{\left\{A_{i}\right\}}_{i=1,2}\right)$ is still missing. Furthermore, the limit does not seem amenable to closed-form characterization. Therefore, we proceed, in the following sections, by deriving *closed-form* analytical lower and upper bounds on this quantity in the large-system limit. These bounds produce, in turn, corresponding inner and outer bounds on the achievable region R which, as implied by (9), are tight in their individual rate constraints.

### 3.2. Inner Bound

**Proposition** **1.** *Let us fix $\kappa_{1}=\kappa \in \left(0,1\right)$, $\kappa_{2}=\bar{\kappa }$, and let $R^{\mathrm{ib}}$ be defined as*

(10) $$\begin{array}{cc} R^{\mathrm{ib}}=\{\left(R_{1},R_{2}\right)\;:R_{1} & \leq C_{1}\left({\mathrm{snr}}_{1}\right),\\ R_{2} & \leq C_{2}\left({\mathrm{snr}}_{2}\right),\\ R_{1}+R_{2} & \leq \log\left(\kappa_{1}2^{C_{1}\left(\frac{{\mathrm{snr}}_{1}}{\kappa_{1}}\right)}+\kappa_{2}2^{C_{2}\left(\frac{{\mathrm{snr}}_{2}}{\kappa_{2}}\right)}\right)\}. \end{array}$$

*Then, the rate region $R^{\mathrm{ib}}$ is achievable for the channel (1) in the large-system limit, as N→∞.*

**Proof.**  See Appendix A. □

As shown in Appendix A, the parameter κ is introduced by a noise-split step required for applying Theorem 1 (see (A2)). The inner bound (10) can then be tightened by *maximizing* the sum-rate constraint over κ∈(0,1) (which can be easily accomplished, e.g., by a straightforward grid-search over (0,1)). As a simple example, consider the extreme case where the receive SNR of either of the user classes vanishes. In such case, the optimized sum-rate constraint can be set arbitrarily close to the actual achievable sum rate. Specifically, assume, without loss of generality, that ${\mathrm{snr}}_{2}\to 0$ (with ${\mathrm{snr}}_{1}$ and κ*fixed*). Clearly, in such case, the achievable throughput of Class 2 users trivially satisfies $R_{2}\to 0$. The sum-rate constraint in (10) then approaches the limit

(11) $$\begin{array}{cc} \log\left(\kappa_{1}2^{C_{1}\left(\frac{{\mathrm{snr}}_{1}}{\kappa_{1}}\right)}+\kappa_{2}2^{C_{2}\left(\frac{{\mathrm{snr}}_{2}}{\kappa_{2}}\right)}\right) & \underset{{\mathrm{snr}}_{2}\to 0}{\to }\log\left(\kappa 2^{C_{1}\left(\frac{{\mathrm{snr}}_{1}}{\kappa }\right)}+1-\kappa \right)\\ \; & \underset{\kappa \to 1}{\to }C_{1}\left({\mathrm{snr}}_{1}\right)\;, \end{array}$$

which is indeed the maximum achievable sum rate in this extreme setting. In fact, the optimization with respect to κ turns out to be rather crucial, since poor choices of κ may lead to cases where not all rate constraints in (10) are active and the inner bound no longer specifies a pentagon in the $\left(R_{1},R_{2}\right)$-plane. As indicated by numerical investigations, this may occur in *underloaded* settings, which are of lesser interest in view of the expected use cases of the underlying NOMA setting.

### 3.3. Outer Bound

**Proposition** **2.** *Let us fix $\mu_{1},\mu_{2}\in \left(0,1\right)$ such that $\mu_{3}≜1-\mu_{1}-\mu_{2}>0$, let ${\bar{\mu }}_{1}≜1-\mu_{1}$, ${\bar{\mu }}_{2}≜1-\mu_{2}$, and let $R^{\mathrm{ob}}$ satisfy*

(12) Rob={(R1,R2):R1≤C1(snr1),Rob={(R2≤C2(snr2),Rob={(R1+R2≤logμ¯1μ¯22C1(snr1μ¯2)+C2(snr2μ¯1)−μ1μ22C1(snr1μ1)+C2(snr2μ2)≤−logμ3}.

*Then, the rate region $R^{\mathrm{ob}}$ includes the achievable region for the channel (1) in the large-system limit, as N→∞.*

**Proof.**  See Appendix B. □

Analogously to the proof of Proposition 1, a noise-split step introducing the parameters $\left\{\mu_{1},\mu_{2}\right\}$ is required for the applicability of Theorem 1 (see (A13)–(A15)) and the outer bound (12) can be tightened by *minimizing* the sum-rate constraint over the choice of $\mu_{1},\mu_{2}\in \left(0,1\right)$, while satisfying $1-\mu_{1}-\mu_{2}>0$. Here as well, a proper choice of $\mu_{1}$ and $\mu_{2}$ is crucial, as, otherwise, the sum-rate constraint may turn out too loose and become inactive.

To obtain more insight, it is also useful to consider the Cover–Wyner region, specifying the ultimate MAC *capacity region* (without any random-spreading constraint). In the current setting, the Cover–Wyner region reads [40]

(13) $$\begin{array}{cc} R^{\mathrm{CW}}=\{\left(R_{1},R_{2}\right):R_{1} & \leq \log(1+\beta_{1}{\mathrm{snr}}_{1})\;,\\ R_{2} & \leq \log(1+\beta_{2}{\mathrm{snr}}_{2})\;,\\ R_{1}+R_{2} & \leq \log(1+\beta_{1}{\mathrm{snr}}_{1}+\beta_{2}{\mathrm{snr}}_{2})\}\;, \end{array}$$

and constitutes a trivial outer bound on the achievable region. A comparison to (13) can thus be used to identify settings where the outer bound of Proposition 2 is indeed useful. Note that (13) is also, in fact, the achievable region with a corresponding *idealized* SCMA scheme, where each user transmits independent Gaussian symbols over each of the utilized orthogonal resources; see, e.g., [20].

### 3.4. Alternative Outer Bound for Symmetric Loading

To complete the characterization of the achievable region, we further introduce an alternative outer bound, which applies to a particular symmetric construction of the signature matrices (henceforth dubbed, for conciseness, *“symmetric construction”*). The bound relies on a simple upper bound on the maximum achievable sum rate $\frac{1}{N}I\left(x_{1},x_{2};y|{\left\{A_{i}\right\}}_{i=1,2}\right)$. Let us consider the case where $K_{1}=K_{2}=\frac{K}{2}$ and thus $\beta_{1}=\beta_{2}=\frac{\beta }{2}$. With some abuse of notation, let us assume now that, in addition to the underlying assumptions of Theorem 1, the signature matrices $A_{1}$ and $A_{2}$ are constructed such that the N×K matrix $A≜[A_{1}\;A_{2}]$ can be associated with the adjacency matrix of a locally tree-like (βd,d)-semiregular bipartite graph A (see Definitions 1 and 2). Additionally, let A have a BGWT with degree distribution $\left(\delta_{\beta d},\delta_{d}\right)$ and parameter $\frac{1}{1+\beta }$ as a weak limit. Analogously to Section 2, we assume here that the pair of matrices $\left(A_{1},A_{2}\right)$ is randomly chosen uniformly and independently per each channel use from the set of matrices satisfying the above properties. Then, the following proposition holds.

**Proposition** **3.** *Let the two user classes have equal sizes, corresponding each to a load $\frac{\beta }{2}$. Let the signature matrices $A_{1}$ and $A_{2}$ be chosen according to the “symmetric construction”. Let $R_{\mathrm{sym}}^{\mathrm{ob}}$ be defined as*

(14) $$\begin{array}{cc} R_{\mathrm{sym}}^{\mathrm{ob}}=\{\left(R_{1},R_{2}\right):R_{1} & \leq C^{\mathrm{opt}}\left({\mathrm{snr}}_{1},\frac{\beta }{2},d\right)\;,\\ R_{2} & \leq C^{\mathrm{opt}}\left({\mathrm{snr}}_{2},\frac{\beta }{2},d\right)\;,\\ R_{1}+R_{2} & \leq C^{\mathrm{opt}}\left(\frac{{\mathrm{snr}}_{1}+{\mathrm{snr}}_{2}}{2},\beta ,d\right)\}\;, \end{array}$$

*where $C^{\mathrm{opt}}\left(\cdot ,\cdot ,d\right)$ is specified in (5). Then, the rate region $R_{\mathrm{sym}}^{\mathrm{ob}}$ includes the achievable region for the channel (1) in the large-system limit, as N→∞.*

**Proof.**  Focusing on the sum-rate constraint, the underlying assumptions of the “symmetric construction” imply that the maximum achievable sum rate satisfies

(15) $$\begin{array}{cc} \frac{1}{N}I\left(x_{1},x_{2};y|{\left\{A_{i}\right\}}_{i=1,2}\right) & =\frac{1}{N}E\left\{\log\left(\det\left(I_{N}+\frac{{\mathrm{snr}}_{1}}{d}A_{1}A_{1}^{†}+\frac{{\mathrm{snr}}_{2}}{d}A_{2}A_{2}^{†}\right)\right)\right\}\\ \; & =\frac{1}{N}E\left\{\log\left(\det\left(I_{N}+A\left(D⊗I_{\frac{K}{2}}\right)A^{†}\right)\right)\right\}\\ \; & \overset{\left(a\right)}{=}\frac{1}{N}E_{A,\Pi }\left\{\log\left(\det\left(I_{N}+A\left(\left(\Pi D\Pi^{T}\right)⊗I_{\frac{K}{2}}\right)A^{†}\right)\right)\right\}\;, \end{array}$$

where

(16) $$D=\frac{1}{d}\left[\begin{array}{cc} {\mathrm{snr}}_{1} & 0\\ 0 & {\mathrm{snr}}_{2} \end{array}\right]\;,$$

Π is a random permutation matrix satisfying

(17) $$\Pi =\left\{\begin{array}{cc} I_{2} & w.p.\;\frac{1}{2}\;,\\ \left[\begin{array}{cc} 0 & 1\\ 1 & 0 \end{array}\right] & w.p.\;\frac{1}{2}\;, \end{array}\right.$$

and (a) follows by symmetry with respect to $A_{1}$ and $A_{2}$. Next, applying Jensen’s inequality, while relying on the convexity of the logdet(·) function, we obtain

(18) 1NI(x1,x2;y|Aii=1,2)1NI(x1,x2;y=1NEAEΠlogdetIN+AΠDΠT⊗IK2A†|A=A1NI(x1,x2;y≤1NEAlogdetIN+AEΠΠDΠT⊗IK2A†1NI(x1,x2;y=1NEAlogdetIN+snravdAA†1NI(x1,x2;y→N→∞Copt(snrav,β,d),

where we define the average SNR as

(19) $${\mathrm{snr}}_{\mathrm{av}}≜\frac{\beta_{1}}{\beta }{\mathrm{snr}}_{1}+\frac{\beta_{2}}{\beta }{\mathrm{snr}}_{2}\;,$$

yielding ${\mathrm{snr}}_{\mathrm{av}}=\frac{{\mathrm{snr}}_{1}+{\mathrm{snr}}_{2}}{2}$ for the “symmetric construction”. The limit in (18) follows from Theorem 1. Combining (18) with (9) finally yields (14), which completes the proof. □

## 4. Extreme-SNR Characterization

To complement the analytical characterization of the achievable rate region by means of the bounds in Propositions 1–3, we focus on the achievable sum rate and provide, in this section, an in-depth investigation of the respective bounds in extreme-SNR regimes. Although all bounds take an explicit closed form, the corresponding expressions are still rather involved. Therefore, the main advantage of the extreme-SNR characterization is that, by means of certain simplifying approximations (appropriate for extreme SNRs), it leads to valuable insights that are otherwise hard to obtain, as is shown in the sequel. In particular, this characterization demonstrates the impact of the parameters $\kappa ,\mu_{1},\mu_{2}$ used in Propositions 1 and 2 on the tightness of the respective lower and upper bounds on the achievable sum rate. For symmetric settings (see Section 3.4), it further allows us to identify which of the two upper bounds on the achievable sum rate stated in Propositions 2 and 3 is tighter; hence, the corresponding outer bound is more useful for characterizing the achievable region in extreme-SNR regimes. For the low-SNR regime, we specify conditions under which the former bound of Proposition 2 is tighter, while, for the high-SNR regime, it turns out that Proposition 3 generally provides a tighter bound in symmetric overloaded settings.

Our analysis examines the achievable sum rate as a function of ${\mathrm{snr}}_{\mathrm{av}}$ (the average SNR), as defined in (19). Furthermore, without loss of generality, we assume henceforth that ${\mathrm{snr}}_{2}=\tilde{\alpha }{\mathrm{snr}}_{1}$ for some $\tilde{\alpha }\in \left(0,1\right)$. This immediately implies (cf. (19)) that

(20) $${\mathrm{snr}}_{1}=\chi {\mathrm{snr}}_{\mathrm{av}}\;,\;{\mathrm{snr}}_{2}=\tilde{\alpha }\chi {\mathrm{snr}}_{\mathrm{av}}\;,$$

where

(21) $$\chi ≜\frac{\beta }{\beta_{1}+\tilde{\alpha }\beta_{2}}\;.$$

Starting with the low-SNR regime, the achievable sum rate is approximated as

(22) $$C_{\mathrm{sum}}\approx \frac{S_{0}}{3\;\mathrm{dB}}\left(\frac{E_{b}}{N_{0}}{{|}_{\mathrm{dB}}-{\left(\frac{E_{b}}{N_{0}}\right)}_{\min}|}_{\mathrm{dB}}\right)\;,$$

where $S_{0}$ denotes the low-SNR slope, ${\left(\frac{E_{b}}{N_{0}}\right)}_{\min}$ is the minimum system average $\frac{E_{b}}{N_{0}}$ that enables reliable communications and $3\;\mathrm{dB}≜10\log_{10}2$ [41]. The average SNR and $\frac{E_{b}}{N_{0}}$ are related via

(23) $$\beta {\mathrm{snr}}_{\mathrm{av}}=C_{\mathrm{sum}}\left({\mathrm{snr}}_{\mathrm{av}}\right)\frac{E_{b}}{N_{0}}\;.$$

Let $C_{\mathrm{sum}}\left({\mathrm{snr}}_{\mathrm{av}}\right)$ denote the achievable sum rate expressed as a function of ${\mathrm{snr}}_{\mathrm{av}}$ in *nats/channel use* per dimension. Then, the minimum $\frac{E_{b}}{N_{0}}$ that enables reliable communications and the low-SNR slope read [41]

(24) $${\left(\frac{E_{b}}{N_{0}}\right)}_{\min}=\frac{\beta \ln2}{{\dot{C}}_{\mathrm{sum}}\left(0\right)}\;,$$

(25) $$S_{0}=-\frac{2{\left[{\dot{C}}_{\mathrm{sum}}\left(0\right)\right]}^{2}}{{\ddot{C}}_{\mathrm{sum}}\left(0\right)}\;,$$

where ${\dot{C}}_{\mathrm{sum}}\left(0\right)$ and ${\ddot{C}}_{\mathrm{sum}}\left(0\right)$ denote the first and second derivatives at zero of $C_{\mathrm{sum}}\left({\mathrm{snr}}_{\mathrm{av}}\right)$ (note that (22), (24) and (25) tacitly assume that the minimum $\frac{E_{b}}{N_{0}}$ corresponds to the point of vanishing rate; a short discussion on this aspect is provided in Appendix D).

Turning to the high-SNR regime, we approximate the achievable sum rate as [41]

(26) $$C_{\mathrm{sum}}\left({\mathrm{snr}}_{\mathrm{av}}\right)\approx S_{\infty }\left(\log{\mathrm{snr}}_{\mathrm{av}}-L_{\infty }\right)\;,$$

where $S_{\infty }$ denotes the high-SNR slope (multiplexing gain) and $L_{\infty }$ denotes the high-SNR power offset. Note that we use here a slightly different high-SNR approximation than the one originally proposed in [41], in the sense that it relies on approximating the sum rate as a function of ${\mathrm{snr}}_{\mathrm{av}}$ (rather than $\frac{E_{b}}{N_{0}}$); consequently, the resulting high-SNR power offset differs by a logβ term when compared to [41].

In the following sections, we employ the above approximations for the lower and upper bounds on the achievable sum rate and derive the corresponding extreme-SNR parameters. Specifically, we consider the sum-rate bounds in (10) and (12), which, when rewritten as functions of ${\mathrm{snr}}_{\mathrm{av}}$, read

(27) $$\begin{array}{cc} C_{\mathrm{sum}}^{\mathrm{lb}}\left({\mathrm{snr}}_{\mathrm{av}}\right) & =\log\left(\kappa_{1}2^{C_{1}\left(\frac{\chi {\mathrm{snr}}_{\mathrm{av}}}{\kappa_{1}}\right)}+\kappa_{2}2^{C_{2}\left(\frac{\tilde{\alpha }\chi {\mathrm{snr}}_{\mathrm{av}}}{\kappa_{2}}\right)}\right)\;, \end{array}$$

(28) $$\begin{array}{cc} C_{\mathrm{sum}}^{\mathrm{ub}}\left({\mathrm{snr}}_{\mathrm{av}}\right) & =\log\left(\frac{{\bar{\mu }}_{1}{\bar{\mu }}_{2}}{{\bar{\mu }}_{12}}2^{C_{1}\left(\frac{\chi {\mathrm{snr}}_{\mathrm{av}}}{{\bar{\mu }}_{2}}\right)+C_{2}\left(\frac{\tilde{\alpha }\chi {\mathrm{snr}}_{\mathrm{av}}}{{\bar{\mu }}_{1}}\right)}-\frac{\mu_{1}\mu_{2}}{{\bar{\mu }}_{12}}2^{C_{1}\left(\frac{\chi {\mathrm{snr}}_{\mathrm{av}}}{\mu_{1}}\right)+C_{2}\left(\frac{\tilde{\alpha }\chi {\mathrm{snr}}_{\mathrm{av}}}{\mu_{2}}\right)}\right)\;, \end{array}$$

where we introduce the notation

(29) $${\bar{\mu }}_{12}≜1-\mu_{1}-\mu_{2}=\mu_{3}\;.$$

For symmetric settings, we additionally rely on ([38], Proposition 5; see also [39], Proposition 4) for the extreme-SNR characterization of the sum-rate bound in (14).

Furthermore, note that the *sum capacity* (specifying the ultimate performance limit) is given by the sum-rate constraint in (13), which, when expressed as a function of ${\mathrm{snr}}_{\mathrm{av}}$, boils down to

(30) $$\begin{array}{cc} C_{\mathrm{sum}}^{\mathrm{CW}}\left({\mathrm{snr}}_{\mathrm{av}}\right)\; & =\log(1+\beta {\mathrm{snr}}_{\mathrm{av}})\;. \end{array}$$

Hence, the corresponding low-SNR parameters are

(31) $${\left(\frac{E_{b}}{N_{0}}\right)}_{\min}^{\mathrm{CW}}=\ln2\;,\;S_{0}^{\mathrm{CW}}=2\;,$$

while the high-SNR parameters read

(32) $$S_{\infty }^{\mathrm{CW}}=1\;,\;L_{\infty }^{\mathrm{CW}}=-\log\beta \;.$$

### 4.1. The low-SNR Regime

Starting with the sum-rate lower bound (27), its low-SNR characterization is summarized in the following proposition.

**Proposition** **4.** *The low-SNR parameters of the asymptotic sum-rate lower bound (27) read*

(33) $$\begin{array}{ccc} {\left(\frac{E_{b}}{N_{0}}\right)}_{\min}^{\mathrm{lb}} & = & \ln2\;, \end{array}$$

(34) $$\begin{array}{ccc} S_{0}^{\mathrm{lb}} & = & \frac{2}{1+\frac{d-1}{d{\left(\beta_{1}+\tilde{\alpha }\beta_{2}\right)}^{2}}\cdot \left(\frac{\beta_{1}}{\kappa_{1}}+\frac{{\tilde{\alpha }}^{2}\beta_{2}}{\kappa_{2}}\right)}\;, \end{array}$$

*where, as in Proposition 1, $\kappa_{1}=\kappa \in \left(0,1\right)$ and $\kappa_{2}=\bar{\kappa }$.*

**Proof.**  See Appendix C. □

As implied by Proposition 4, the sum-rate lower bound optimization with respect to κ (see Section 3.2) takes a more explicit form in the low-SNR regime. Specifically, the low-SNR slope (34) can be optimized by choosing κ∈(0,1) such that the denominator therein is minimized; namely, by setting

(35) $$\kappa =\kappa_{\mathrm{opt}}^{L}≜\underset{\kappa \in (0,1)}{arg min}\frac{\beta_{1}}{\kappa }+\frac{{\tilde{\alpha }}^{2}\beta_{2}}{1-\kappa }\;.$$

To demonstrate the usefulness of this observation, while simplifying the discussion, let us consider the symmetric setting where $\beta_{1}=\beta_{2}=\frac{\beta }{2}$. Then, the optimal choice for κ reduces to

(36) $$\underset{\kappa \in (0,1)}{arg min}\frac{1}{\kappa }+\frac{{\tilde{\alpha }}^{2}}{1-\kappa }\;,$$

which yields

(37) $$\kappa_{\mathrm{opt}}^{L}=\frac{1}{1+\tilde{\alpha }}\;,\;\tilde{\alpha }\in \left(0,1\right)\;.$$

Substituting back into (34), while accounting for the class-symmetry and setting

(38) $$\kappa_{1}=\frac{1}{1+\tilde{\alpha }}\;,\;\kappa_{2}=1-\kappa_{1}=\frac{\tilde{\alpha }}{1+\tilde{\alpha }}\;,$$

we obtain (following some algebra)

(39) $$\begin{array}{cc} S_{0}^{\mathrm{lb}}\; & =\frac{2\beta d}{\beta d+2(d-1)}\;. \end{array}$$

Note that $S_{0}^{\mathrm{lb}}$ is strictly lower, for all d≥2, than the corresponding low-SNR slope of the optimum spectral efficiency (total throughput) in a *single-class setting* with load β and an SNR that equals ${\mathrm{snr}}_{\mathrm{av}}$, as specified in Theorem 1, which reads ([38], Proposition 5; see also [39], Proposition 4)

(40) $$S_{0}^{\mathrm{SC}}=\frac{2\beta d}{d(\beta +1)-1}=\frac{2\beta d}{\beta d+d-1}\;.$$

In fact, (40) also specifies the low-SNR slope of the sum-rate upper bound for the “symmetric construction”, as stated in Proposition 3 (cf. (14)). Hence, (39) and (40) provide compact lower and upper bounds on the low-SNR slope of the achievable sum rate under class symmetry. Another interesting comparison is with the low-SNR slope of the maximum achievable sum rate with RS-CDMA, representing a practical manifestation of random *dense* NOMA (see Appendix G for a derivation of the RS-CDMA achievable region). For the symmetric setting, this slope reads (following (A110), (A114) and ([41], Equation (147)))

(41) $$\begin{array}{cc} S_{0}^{\mathrm{RS}}\; & =\frac{2}{1+\frac{2(1+{\tilde{\alpha }}^{2})}{\beta {\left(1+\tilde{\alpha }\right)}^{2}}}=\frac{2\beta }{\beta +\frac{2(1+{\tilde{\alpha }}^{2})}{{\left(1+\tilde{\alpha }\right)}^{2}}}\;, \end{array}$$

which lets us conclude that $S_{0}^{\mathrm{lb}}>S_{0}^{\mathrm{RS}}$, as long as

(42) $$\frac{d-1}{d}<\frac{1+{\tilde{\alpha }}^{2}}{{\left(1+\tilde{\alpha }\right)}^{2}}\;,$$

hence, regular sparse NOMA is guaranteed to strictly outperform RS-CDMA in the low-SNR regime as long as (42) is satisfied. Note, e.g., that setting d=2 immediately implies that (42) is satisfied for all $\tilde{\alpha }\in \left(0,1\right)$.

Turning to the sum-rate upper bound (28), its low-SNR characterization is summarized in the following proposition.

**Proposition** **5.** *Let D denote the set*

(43) $$\begin{array}{c} D≜\left\{\mu_{1},\mu_{2}:\mu_{1},\mu_{2}\in \left(0,1\right)\;,\;1-\mu_{1}-\mu_{2}>0\;,\;\left(\mu_{1}-\mu_{2}\right)\left(\frac{\beta_{1}}{\mu_{1}{\bar{\mu }}_{2}}-\frac{{\tilde{\alpha }}^{2}\beta_{2}}{\mu_{2}{\bar{\mu }}_{1}}\right)>0\right\}\;, \end{array}$$

*where, as in Proposition 2, ${\bar{\mu }}_{1}≜1-\mu_{1}$ and ${\bar{\mu }}_{2}≜1-\mu_{2}$. Then, for $(\mu_{1},\mu_{2})\in D$, the low-SNR parameters of the asymptotic sum-rate upper bound (28) read*

(44) $$\begin{array}{ccc} {\left(\frac{E_{b}}{N_{0}}\right)}_{\min}^{\mathrm{ub}} & = & \ln2\;, \end{array}$$

(45) $$\begin{array}{ccc} S_{0}^{\mathrm{ub}} & = & \frac{2}{1+\frac{\left(\mu_{1}-\mu_{2}\right)\left(d-1\right)}{d{\left(\beta_{1}+\tilde{\alpha }\beta_{2}\right)}^{2}}\left(\frac{\beta_{1}}{\mu_{1}{\bar{\mu }}_{2}}-\frac{{\tilde{\alpha }}^{2}\beta_{2}}{\mu_{2}{\bar{\mu }}_{1}}\right)}\;. \end{array}$$

*Furthermore, the sum-rate upper bound (28) is not useful in the low-SNR regime for $(\mu_{1},\mu_{2})\notin \;D$.*

**Proof.**  See Appendix D. □

Analogously to the characterization of the lower bound in Proposition 4, the low-SNR slope (45) can be optimized by choosing $\left(\mu_{1},\mu_{2}\right)$ such that the denominator therein is maximized; namely, by setting

(46) $$\left(\mu_{1},\mu_{2}\right)=\left(\mu_{1,\mathrm{opt}}^{U},\mu_{2,\mathrm{opt}}^{U}\right)≜\underset{(\mu_{1},\mu_{2})\in D}{arg max}\left(\mu_{1}-\mu_{2}\right)\left(\frac{\beta_{1}}{\mu_{1}{\bar{\mu }}_{2}}-\frac{{\tilde{\alpha }}^{2}\beta_{2}}{\mu_{2}{\bar{\mu }}_{1}}\right)\;.$$

Note that, for $(\mu_{1},\mu_{2})\in D$, the low-SNR slope $S_{0}^{\mathrm{ub}}$ is strictly smaller than $S_{0}^{\mathrm{CW}}=2$ (recall that d≥2); hence, the upper bound (28) is useful in this region of the parameters.

Additional insight can be gained by focusing again on the symmetric setting $\beta_{1}=\beta_{2}=\frac{\beta }{2}$. In such case, we obtain that the optimal choice for $\left(\mu_{1},\mu_{2}\right)$ simplifies to the following:

(47) $$\begin{array}{cc} (\mu_{1},\mu_{2})\; & =\underset{(\mu_{1},\mu_{2})\in D}{arg max}\frac{2\left(\mu_{1}-\mu_{2}\right)\left(d-1\right)}{\beta d{\left(1+\tilde{\alpha }\right)}^{2}}\left(\frac{1}{\mu_{1}{\bar{\mu }}_{2}}-\frac{{\tilde{\alpha }}^{2}}{\mu_{2}{\bar{\mu }}_{1}}\right)\\ \; & =\underset{(\mu_{1},\mu_{2})\in D}{arg max}\left(\mu_{1}-\mu_{2}\right)\left(\frac{1}{\mu_{1}{\bar{\mu }}_{2}}-\frac{{\tilde{\alpha }}^{2}}{\mu_{2}{\bar{\mu }}_{1}}\right)\;. \end{array}$$

Note that, in fact, for $\beta_{1}=\beta_{2}=\frac{\beta }{2}$, the condition that specifies the set D simplifies to $\left(\mu_{1}-\mu_{2}\right)\left(\frac{1}{\mu_{1}{\bar{\mu }}_{2}}-\frac{{\tilde{\alpha }}^{2}}{\mu_{2}{\bar{\mu }}_{1}}\right)>0$. Then, rewriting the low-SNR slope (45) as

(48) $$\begin{array}{cc} S_{0}^{\mathrm{ub}} & =\frac{2}{1+\frac{2\left(\mu_{1}-\mu_{2}\right)\left(d-1\right)}{\beta d{\left(1+\tilde{\alpha }\right)}^{2}}\left(\frac{1}{\mu_{1}{\bar{\mu }}_{2}}-\frac{{\tilde{\alpha }}^{2}}{\mu_{2}{\bar{\mu }}_{1}}\right)}\\ \; & =\frac{2\beta d}{\beta d+\left(d-1\right)\cdot \frac{2(\mu_{1}-\mu_{2})}{{\left(1+\tilde{\alpha }\right)}^{2}}\left(\frac{1}{\mu_{1}{\bar{\mu }}_{2}}-\frac{{\tilde{\alpha }}^{2}}{\mu_{2}{\bar{\mu }}_{1}}\right)}\;, \end{array}$$

we conclude that the sum-rate upper bound (28) implied by Proposition 2 is *tighter* in the low-SNR regime than the corresponding simple “symmetric construction“ bound in (14), as long as there exists a pair of constants $(\mu_{1},\mu_{2})\in D$ such that (cf. (40))

(49) $$\frac{2(\mu_{1}-\mu_{2})}{{\left(1+\tilde{\alpha }\right)}^{2}}\left(\frac{1}{\mu_{1}{\bar{\mu }}_{2}}-\frac{{\tilde{\alpha }}^{2}}{\mu_{2}{\bar{\mu }}_{1}}\right)>1\;.$$

Note that the existence of such constants is not guaranteed for all choices of the underlying parameters. To see this, let $\tau ≜\frac{\mu_{2}}{\mu_{1}}$ and let us recall that, since $1-\mu_{1}-\mu_{2}>0$, the ratio τ must satisfy $0<\tau <\frac{1}{\mu_{1}}-1$. Then, (49) can be rewritten as

(50) $$\begin{array}{cc} \frac{2(1-\tau )}{{\left(1+\tilde{\alpha }\right)}^{2}}\left(\frac{1}{1-\tau \mu_{1}}-\frac{{\tilde{\alpha }}^{2}}{\tau (1-\mu_{1})}\right) & >1\;, \end{array}$$

or, equivalently,

(51) $$\frac{A\tau^{2}+B\tau +C}{\tau \left(1-\tau \mu_{1}\right)\left(1-\mu_{1}\right){\left(1+\tilde{\alpha }\right)}^{2}}>0\;,$$

where $\tau \left(1-\tau \mu_{1}\right)\left(1-\mu_{1}\right){\left(1+\tilde{\alpha }\right)}^{2}>0$ and

(52) $$\begin{array}{ccc} A & = & -{\left(1+\tilde{\alpha }\right)}^{2}\mu_{1}^{2}+\left(3-\tilde{\alpha }\right)\left(1+\tilde{\alpha }\right)\mu_{1}-2\;, \end{array}$$

(53) $$\begin{array}{ccc} B & = & \left(1+\tilde{\alpha }\right)\left(3\tilde{\alpha }-1\right)\mu_{1}+{\left(1-\tilde{\alpha }\right)}^{2}\;, \end{array}$$

(54) $$\begin{array}{ccc} C & = & -2{\tilde{\alpha }}^{2}\;. \end{array}$$

Now, a careful examination of the constants A, B and C reveals that a *necessary* condition for (51) to hold is

(55) $$0<\tilde{\alpha }<3-2\sqrt{2}\approx 0.1716\;.$$

That is, the sum-rate upper bound (28) can be tighter than the corresponding “symmetric construction“ upper bound in (14) only if $\tilde{\alpha }$ is relatively low. To determine *sufficiency*, let us consider the polynomial $P\left(\tau \right)≜A\tau^{2}+B\tau +C$. Then, it can be verified that, subject to condition (55), the coefficients A, B and C satisfy

(56) $$A<0\;,\;B>0\;,C<0\;,\;\mu_{1}\in \left(0,1\right)\;,\;0<\tilde{\alpha }<3-2\sqrt{2}\;.$$

Let $\tau_{1}$ and $\tau_{2}$ denote the roots of P(τ), namely,

(57) $$\tau_{1}=\frac{-B+\sqrt{B^{2}-4AC}}{2A}\;,\;\tau_{2}=\frac{-B-\sqrt{B^{2}-4AC}}{2A}\;.$$

Then (noting that $B^{2}-4AC>0$),

(58) $$\tau_{1}<\tau_{2}\;,\;\mu_{1}\in \left(0,1\right)\;,\;0<\tilde{\alpha }<3-2\sqrt{2}\;,$$

and it turns out that, in addition to (55), condition (51) can only hold if the following additional conditions are satisfied:

(59) $$\left\{\begin{array}{cc} \tau_{1}<\tau <\tau_{2}\;, & \mathrm{If}\;\tau_{2}<\frac{1}{\mu_{1}}-1\;,\\ \tau_{1}<\tau <\frac{1}{\mu_{1}}-1\;, & \mathrm{If}\;\tau_{1}<\frac{1}{\mu_{1}}-1<\tau_{2}\;. \end{array}\right.$$

### 4.2. The High-SNR Regime

As in Section 4.1, we start with the sum-rate lower bound (27) and derive its high-SNR characterization while focusing on the case where at least one of the two user classes is overloaded. For concreteness, we assume henceforth that $\beta_{1}>1$. Our main observations are summarized in the following proposition.

**Proposition** **6.** *Assume that $\beta_{1}>1$. Then, the high-SNR parameters of the asymptotic sum-rate lower bound (27) read*

(60) $$S_{\infty }^{\mathrm{lb}}=1$$

*and*

(61) $$L_{\infty }^{\mathrm{lb}}=\left\{\begin{array}{cc} L_{\infty ,1}+L_{\infty ,2}-\log\left(2^{L_{\infty ,1}}+\frac{1}{\tilde{\alpha }}2^{L_{\infty ,2}}\right)-\log\left(\tilde{\alpha }\chi \right) & ,\;\beta_{2}\geq 1\\ L_{\infty ,1}-\log\chi & ,\;\frac{2}{d}\leq \beta_{2}<1 \end{array}\right.$$

*where $L_{\infty ,i}$, i=1,2, denotes the high-SNR power offset of a* single-class *setting with load $\beta_{i}$ (corresponding to Theorem 1), which reads ([38], Proposition 5)*

(62) $$L_{\infty ,i}=\left\{\begin{array}{cc} \left(\frac{1}{\beta_{i}}-1\right)\log\left(1-\beta_{i}\right)-\left(d-1\right)\log\left(1-\frac{1}{d}\right), & \beta_{i}<1\\ -\left(d-1\right)\log\left(1-\frac{1}{d}\right), & \beta_{i}=1\\ \left(\beta_{i}-1\right)\log\left(\beta_{i}-1\right)-\beta_{i}\log\beta_{i}-\left(\beta_{i}d-1\right)\log\left(1-\frac{1}{\beta_{i}d}\right), & \beta_{i}>1\;. \end{array}\right.$$

**Proof.**  See Appendix E. □

**Remark** **1.** *Analogous results for the case where the load $\beta_{2}$ is constrained to satisfy $\beta_{2}>1$, as well as the case where $\beta_{1}=\beta_{2}=1$, can be straightforwardly derived following similar lines to the proof in Appendix E. The details are omitted for conciseness. Note also that, for the case where* both *user classes are underloaded (i.e., $\frac{2}{d}\leq \beta_{1},\beta_{2}<1$), the corresponding derivation indicates that the sum-rate lower bound is too loose in the high-SNR regime and fails to capture the correct high-SNR slope.*

**Remark** **2.** *As indicated by (60) and (61), in sheer contrast to the low-SNR regime, the high-SNR parameters of the sum-rate lower bound* do not *depend on the parameter κ for $\beta_{1}>1$.*

The high-SNR characterization of the sum-rate upper bound (28) is summarized next.

**Proposition** **7.** *Assume that $\beta_{1},\beta_{2}>1$. Then, the high-SNR parameters of the asymptotic sum-rate upper bound (28) read*

(63) $$S_{\infty }^{\mathrm{ub}}=1\;,$$

*and*

(64) $$L_{\infty }^{\mathrm{ub}}=L_{\infty ,1}+L_{\infty ,2}-\log\left(\left(\frac{\left(\beta_{1}d-1\right)\tilde{\alpha }}{\beta_{1}\left(\beta_{1}-1\right)}+\frac{\beta_{2}d-1}{\beta_{2}\left(\beta_{2}-1\right)}\right)\cdot \frac{\chi }{d}\right)\;,$$

*where $L_{\infty ,i}$, i=1,2, are specified in (62).*

**Proof.**  See Appendix F. □

**Remark** **3.** *Note that, for $\beta_{1},\beta_{2}>1$, the high-SNR parameters of the sum-rate upper bound turn out to be independent of the choice of $\mu_{1},\mu_{2}$. Derivations along the lines of Appendix F reveal that, for $\frac{2}{d}\leq \beta_{1},\beta_{2}<1$, the sum-rate upper bound follows the correct high-SNR slope only if $\beta_{1}+\beta_{2}\leq 1$, namely, when the (overall) system operates in the underloaded (or fully loaded) regime, which is of lesser interest in the NOMA framework. Furthermore, the analysis shows that, for all other choices of $\beta_{1}$ and $\beta_{2}$ (expect for $\beta_{1},\beta_{2}>1$), the sum-rate upper bound (28) becomes too loose for the high-SNR regime and does not capture the correct high-SNR slope.*

To gain more insight, let us consider the symmetric overloaded setting where $\beta_{1}=\beta_{2}=\frac{\beta }{2}>1$. In such case, the high-SNR slope equals unity for both the lower and upper sum-rate bounds, namely,

(65) $$S_{\infty }^{\mathrm{lb}}=S_{\infty }^{\mathrm{ub}}=1\;.$$

Turning to the high-SNR power offset, note that, in the symmetric overloaded setting, one obtains (cf. (21))

(66) $$\chi =\frac{2}{1+\tilde{\alpha }},$$

while

(67) $$\begin{array}{cc} L_{\infty ,1} & =L_{\infty ,2}\\ \; & =\frac{\beta -2}{2}\log\left(\beta -2\right)-\frac{\beta }{2}\log\left(\beta \right)-\left(\frac{\beta d-2}{2}\right)\log\left(1-\frac{2}{\beta d}\right)+1\;. \end{array}$$

Hence, we conclude, from (61) and (67), that

(68) $$\begin{array}{cc} L_{\infty }^{\mathrm{lb}}\; & =L_{\infty ,1}-1\\ \; & =\frac{\beta -2}{2}\log\left(\beta -2\right)-\frac{\beta }{2}\log\beta -\left(\frac{\beta d-2}{2}\right)\log\left(1-\frac{2}{\beta d}\right)\;. \end{array}$$

Similarly, considering the high-SNR power offset of the sum-rate upper bound (64), we obtain

(69) $$\begin{array}{cc} L_{\infty }^{\mathrm{ub}}\; & =2L_{\infty ,1}+\log\left(\beta -2\right)-\log\left(1-\frac{2}{\beta d}\right)-2\\ \; & =\left(\beta -1\right)\log\left(\beta -2\right)-\beta \log\left(\beta \right)-\left(\beta d-1\right)\log\left(1-\frac{2}{\beta d}\right)\;. \end{array}$$

The difference between the offsets (68) and (69) specifies the horizontal gap (in logarithmic scale) between these lower and upper bounds on the achievable sum rate in the high-SNR regime. However, it is also insightful to compare $L_{\infty }^{\mathrm{ub}}$ to the corresponding high-SNR power offset induced by the “symmetric construction” outer bound of Proposition 3 (see (14)). This offset is simply given by (62), while substituting β instead of $\beta_{i}$, and reads

(70) $$L_{\infty ,\mathrm{sym}}^{\mathrm{ub}}=\left(\beta -1\right)\log\left(\beta -1\right)-\beta \log\beta -\left(\beta d-1\right)\log\left(1-\frac{1}{\beta d}\right)\;.$$

Note that

(71) $$\begin{array}{cc} L_{\infty }^{\mathrm{ub}}-L_{\infty ,\mathrm{sym}}^{\mathrm{ub}}\; & =\left(\beta -1\right)\log\left(\frac{\beta -2}{\beta -1}\right)+\left(\beta d-1\right)\log\left(\frac{\beta d-1}{\beta d-2}\right)\;, \end{array}$$

which can be verified to always take on negative values for β>2 and d≥2 (in fact, in such case, $L_{\infty }^{\mathrm{ub}}$ is even strictly smaller than $L_{\infty }^{\mathrm{CW}}$ (32)). Hence, we conclude that the “symmetric construction” sum-rate upper bound is tighter in the high-SNR regime for symmetric settings, when $\beta_{1}=\beta_{2}>1$. This observation is corroborated by the numerical results presented in Section 5.

## 5. Numerical Results

In this section, we present some numerical results that demonstrate the effectiveness of the inner and outer bounds derived in Section 3 for assessing the potential performance gains of regular sparse NOMA. The focus here is on *overloaded* settings ($\beta_{1},\beta_{2}>1$), corresponding to use cases where NOMA is of particular interest, while noting that the bounds also generally apply to underloaded regimes. The sparsity parameter *d* of the signature matrices was set throughout to d=2, since, as shown in [38,39], it induces the highest achievable individual (per class) throughputs for regular sparse NOMA. Our numerical investigation complements the *analytical* observations of Section 4, by considering more general SNR regimes.

**Figure 1:**
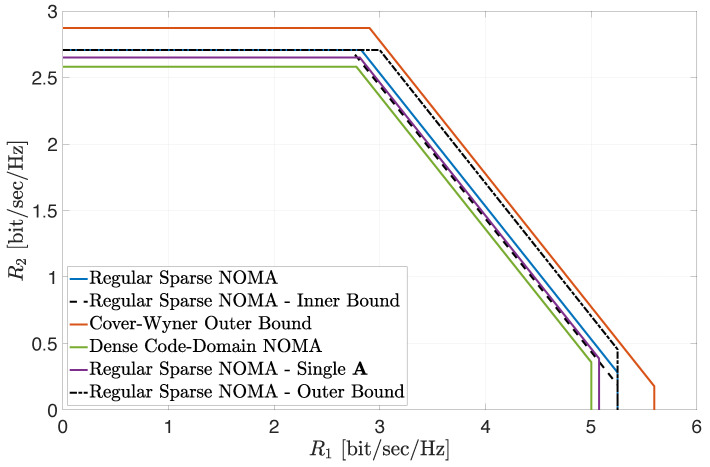
Achievable regions for ${\mathrm{snr}}_{1}=15\;\mathrm{dB}$, ${\mathrm{snr}}_{2}=5\;\mathrm{dB}$, d=2, $\beta_{1}=1.5$ and $\beta_{2}=2$.

Figure 1 depicts the inner and outer bounds on the achievable region (8) in the large-system limit, for the case where $\beta_{1}=1.5$ and $\beta_{2}=2$ (cf. (10) and (12)). The corresponding noise-split parameters were set to κ=0.9, $\mu_{1}=0.05$ and $\mu_{2}=0.9$ (these values were numerically verified to be close to optimal). The SNRs of the two user classes were fixed to ${\mathrm{snr}}_{1}=15\;\mathrm{dB}$ and ${\mathrm{snr}}_{2}=5\;\mathrm{dB}$. The cautious reader should note that this choice for the noise split parameters $\left(\mu_{1},\mu_{2}\right)$ is, by no means, a contradiction to the condition specified in Proposition 5 for the usefulness of the outer bound (see (43)). This is since Proposition 5 only applies to the low-SNR regime, while, in Figure 1, the two SNRs *do not* yield a low average SNR setting. In fact, it can be numerically verified that choosing $\left(\mu_{1},\mu_{2}\right)=\left(0.05,0.9\right)$ in the low-SNR regime, which falls outside the region D in (43), leads to a very loose upper bound on the achievable sum rate, significantly higher than the corresponding Cover–Wyner sum-rate upper bound in (13). Hence, this choice is not useful in the low-SNR regime as predicted by Proposition 5 (see also the discussion in Section 4.1). The boundary of the inner bound is represented Figure 1 by the dashed black line, while the boundary of the outer bound is designated by the dash–dotted black line. Note that the two bounds differ only in the sum-rate constraint, while sharing the class–individual rate constraints (which are characterized in full via Theorem 1, as discussed in Section 3.1). To assess the tightness of the bounds, Figure 1 also shows an estimation of the boundary of the achievable region (8) for a regular sparse NOMA system with a large but *finite* number of orthogonal dimensions N=30. Here, all rate constraints of the corresponding region were evaluated based on Monte Carlo (MC) simulations of 1000 sparse matrix realizations using Gallager’s construction for parity-check matrices of LDPC codes [38,39,53] (let us recall that the main motivation for the derivation of the inner and outer bounds on the achievable region is the fact that an exact analytical asymptotic result for the sum-rate constraint of this region is *still lacking*; hence, we resorted to MC simulations). The boundary of this region is represented by the solid blue line. Note that both inner and outer bounds are rather tight and provide a very good assessment of the achievable region (specifically, the limiting upper bound on the sum-rate constraint is higher than the simulated constraint by about only 3%, while the corresponding lower bound is lower by less than 2%). Figure 1 also indicates that the asymptotic class–individual rate constraints provide an *extremely tight* assessment of the corresponding throughputs in large finite-dimensional systems, as already shown in [38,39]. We further note that extensive numerical investigations indicate that the EPI-based bounds become tighter as the powers allocated to each class of users become more unbalanced, which stems from the nature of the EPIs. This observation is further demonstrated in the sequel.

For the sake of comparison, Figure 1 also includes the achievable regions of two other system settings of interest. The first region corresponds to dense code-domain NOMA, represented here by RS-CDMA. The corresponding boundary of the achievable region in the large-system limit is designated by the solid green line (see (A119) in Appendix G). The second region corresponds to a setting where the signatures of individual users are taken as the columns of a *single regular sparse matrix*, where each user occupies d=2 resources and each resource is utilized by exactly $\beta d=(\beta_{1}+\beta_{2})d$ users. No regularity is imposed within either of the two classes of users and the signature columns are allocated to the users in each class uniformly at random. The induced signature matrices $A_{1}$ and $A_{2}$ for each class of users (after reordering of the columns) are therefore *no longer row-regular*. The boundary of the corresponding achievable region, based on MC simulations (while fixing N=30), is designated by the solid purple line (referred to as ‘Single A’). Finally, Figure 1 also shows the Cover–Wyner region (13). The boundary of this region is designated by the solid red line.

A comparison of all achievable regions in Figure 1 lets us conclude the following (with the aid of additional investigations omitted for conciseness): Irregularity induces a loss in the achievable rate region. This is clearly observed when comparing the achievable region with a *single* regular sparse signature matrix construction (‘Single A’) and the MC-based achievable region with two separate regular constructions of the signature matrices for the two classes of users. The superiority of separate constructions is also observed for the *class–individual* rate constraints when considering the inner bound (10). Comparing the regular sparse construction in (1) and dense code-domain NOMA (here, RS-CDMA), the MC simulation results indicate that the corresponding achievable region strictly includes the achievable region with dense code-domain NOMA, in full agreement with the analytical observations made in [38,39] with respect to single-class systems. Furthermore, when the class-powers are far enough apart, this even holds for the inner bound (10), as shown in Figure 1, implying that regular sparse NOMA indeed strictly outperformed dense code-domain NOMA (RS-CDMA) in this setting. Finally, considering the outer bound on the achievable region (12), Figure 1 shows that, for power-unbalanced settings, it provides a good assessment of the gap to the ultimate performance limits, as designated by the Cover–Wyner capacity region.

**Figure 2:**
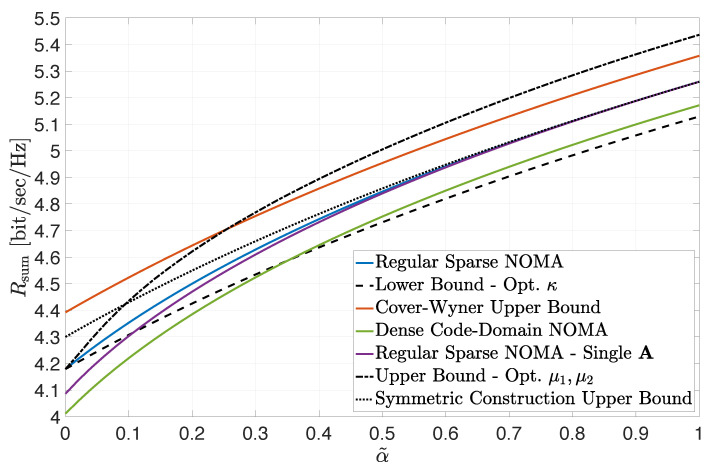
Achievable sum rate for ${\mathrm{snr}}_{1}=10\;\mathrm{dB}$, ${\mathrm{snr}}_{2}=\tilde{\alpha }{\mathrm{snr}}_{1}$ ($\tilde{\alpha }\in \left(0,1\right)$), d=2, $\beta_{1}=2$ and $\beta_{2}=2$. Bounds are plotted for numerically optimized κ, $\mu_{1}$, $\mu_{2}$.

The impact of the SNR balance between the two classes of users is illustrated in Figure 2. A load-symmetric setting is considered where $\beta_{1}=\beta_{2}=2$ and the received SNR of Class 1 users was set to ${\mathrm{snr}}_{1}=10\;\mathrm{dB}$. The received SNR of Class 2 users was set to ${\mathrm{snr}}_{2}=\tilde{\alpha }{\mathrm{snr}}_{1}$, where $\tilde{\alpha }\in \left(0,1\right)$. The figure depicts the sum-rate constraint of the rate region for the settings and bounds considered in Figure 1 as a function of $\tilde{\alpha }$. The lower and upper bounds, according to (10) and (12), were numerically optimized, respectively, by fine-tuning the values of κ, $\mu_{1}$ and $\mu_{2}$. In view of the load-symmetry in this example, the “symmetric construction” upper bound in (14) is also included in the figure (designated by the dotted line). Similar conclusions to the ones discussed with respect to Figure 1 can be reached. The tightness of all three bounds is clearly demonstrated in the low $\tilde{\alpha }$ region. In particular, note that a threshold value for $\tilde{\alpha }$ is observed, below which the sum-rate lower bound in (10) already surpasses the dense code-domain NOMA achievable sum rate (thus guaranteeing the superiority of regular sparse NOMA). The results also indicate that the sum-rate upper bound in (12) is meaningful and resides below the Cover–Wyner sum capacity, when $\tilde{\alpha }$ lies below a threshold (while, otherwise, it becomes too loose and ceases to be useful). Note that, in this load-symmetric setting, the simple “symmetric construction” upper bound in (14) turns out to be the tightest for most values of $\tilde{\alpha }$. In fact, by Jensen’s inequality, it also provides here a tight upper bound for the ‘Single A’ construction. However, neither of the two upper bounds (in (12) or (14)) is universally superior. Although Figure 2 does not represent an extreme-SNR regime per se, the main observations *qualitatively* corroborate the conclusions of the analytical examination in Section 4.

**Figure 3:**
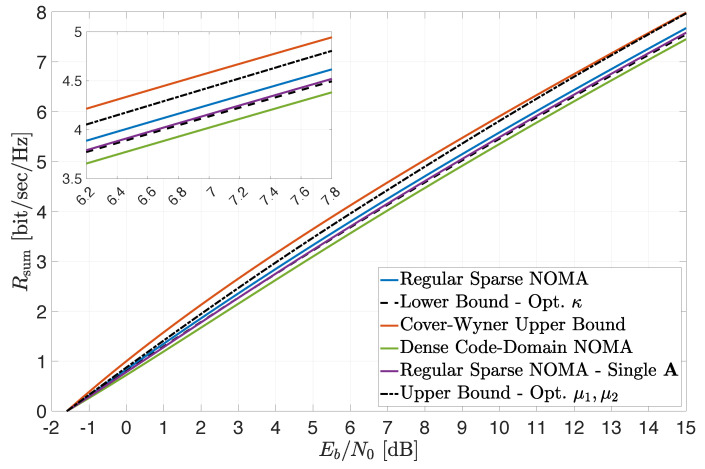
Achievable sum rate for ${\mathrm{snr}}_{2}=0.1\times {\mathrm{snr}}_{1}$, d=2, $\beta_{1}=1.5$ and $\beta_{2}=2$. Bounds are plotted for numerically optimized κ, $\mu_{1}$, $\mu_{2}$.

Finally, the preceding observations are further corroborated in Figure 3, where a setting with $\beta_{1}=1.5$, $\beta_{2}=2$ and ${\mathrm{snr}}_{2}=0.1\times {\mathrm{snr}}_{1}$ is considered (similarly to Figure 1). Here, the sum-rate constraints depicted in Figure 2 (excluding the “symmetric construction” upper bound (14)) are plotted as a function of the system average $\frac{E_{b}}{N_{0}}$ (see (19) and (23)). The tightness of the derived bounds over a wide range of $\frac{E_{b}}{N_{0}}$ values is clearly demonstrated, as well as the superior performance of regular sparse NOMA compared to dense code-domain NOMA (which yields lower sum rates for all $\frac{E_{b}}{N_{0}}$ values). Again, the results are in full agreement with the conclusions of Section 4 regarding extreme-SNR regimes.

## 6. Conclusions

We investigate, in this paper, the achievable rate region in the large-system limit of regular sparse NOMA with two user classes. The analytical challenges induced by the underlying random matrix model were circumvented by deriving *closed-form* inner and outer bounds on the achievable region. These bounds provide a valuable assessment of the potential performance gains of regular sparse NOMA in use cases of interest beyond the equal-power setting previously considered in [38,39]. The superiority of regular sparse NOMA compared with highly complex dense alternatives is demonstrated. The analytical characterization of the bounds in extreme-SNR regimes further elucidates their usefulness, while exhibiting the benefits of their closed-form expressions.

It is important to recall, at this point, that regular sparse NOMA requires *fully coordinated* signatures (to retain the regular structure of the signature matrices); therefore, it might be inapplicable to massive connectivity use cases with sporadic user activity, where such coordination cannot be practically accomplished. Hence, to complement the current analysis, a similar setting with two *mixed* classes of users, where one class employs regular sparse NOMA and the second class employs random dense NOMA, was recently considered in [54]. For this representation of a mixture of fully coordinated and lightly coordinated users, the achievable region was completely and *rigorously* characterized using fundamental tools from free probability theory [30,55]. Potential extensions of both lines of work to account for multiple user classes and multicell networks, as well as the impact of fading and additional channel impairments, are the subject of current and future planned investigations.

## Data Availability

Not applicable.

## Abbreviations

The following abbreviations are used in this manuscript:

AWGN	Additive white Gaussian noise
BGWT	Bipartite Galton–Watson tree
CDMA	Code-division multiple access
EPI	Entropy power inequality
GWT	Galton–Watson tree
i.i.d.	Independent identically distributed
LDCD	Low-density code-domain
LDPC	Low-density parity-check
MAC	Multiple access channel
MC	Monte Carlo
MPA	Message-passing algorithm
MUD	Multiuser detection
NOMA	Non-orthogonal multiple access
RHS	Right-hand side
RS	Randomly spread
SIC	Successive interference cancellation
SNR	Signal-to-noise ratio

## Appendix A. Proof of Proposition 1

By the fundamental properties of mutual information, the sum-rate constraint in (8) can be decomposed as

(A1) $$\begin{array}{cc} \frac{1}{N}I\left(x_{1},x_{2};y|{\left\{A_{i}\right\}}_{i=1,2}\right)\; & =\frac{1}{N}h\left(y|{\left\{A_{i}\right\}}_{i=1,2}\right)-\frac{1}{N}h\left(y|x_{1},x_{2},{\left\{A_{i}\right\}}_{i=1,2}\right)\\ \; & =\frac{1}{N}h\left(y|{\left\{A_{i}\right\}}_{i=1,2}\right)-\frac{1}{N}h\left(z\right)\\ \; & =\frac{1}{N}h\left(y|{\left\{A_{i}\right\}}_{i=1,2}\right)-\log\left(\pi e\right)\;. \end{array}$$

Now, let us fix κ∈(0,1) and let $\kappa_{1}=\kappa$, $\kappa_{2}=\bar{\kappa }=1-\kappa$. Then, the channel output y (cf. (1)) is *statistically* equivalent to

(A2) $$\tilde{y}=\sqrt{\frac{{\mathrm{snr}}_{1}}{d}}A_{1}x_{1}+{\tilde{z}}_{1}+\sqrt{\frac{{\mathrm{snr}}_{2}}{d}}A_{2}x_{2}+{\tilde{z}}_{2}=w_{1}+w_{2}\;,$$

where ${\tilde{z}}_{1}∼CN\left(0,\kappa_{1}I_{N}\right)$, ${\tilde{z}}_{2}∼CN\left(0,\kappa_{2}I_{N}\right)$ and ${\tilde{z}}_{1}$ and ${\tilde{z}}_{2}$ are independent. The vectors

(A3) $$w_{i}≜\sqrt{\frac{{\mathrm{snr}}_{i}}{d}}A_{i}x_{i}+{\tilde{z}}_{i}\;,\;i=1,2\;,$$

are thus statistically independent conditional on the signature matrices ${\left\{A_{i}\right\}}_{i=1,2}$ and have a corresponding multivariate complex Gaussian density. Hence, since

(A4) $$\frac{1}{N}h\left(y|{\left\{A_{i}\right\}}_{i=1,2}\right)=\frac{1}{N}h\left(\tilde{y}|{\left\{A_{i}\right\}}_{i=1,2}\right)\;,$$

it follows, by the *conditional EPI* [43] and (A2), that

(A5) $$\begin{array}{cc} 2^{\frac{1}{N}h\left(y|{\left\{A_{i}\right\}}_{i=1,2}\right)} & =2^{\frac{1}{N}h\left(\tilde{y}|{\left\{A_{i}\right\}}_{i=1,2}\right)}\geq 2^{\frac{1}{N}h\left(w_{1}|{\left\{A_{i}\right\}}_{i=1,2}\right)}+2^{\frac{1}{N}h\left(w_{2}|{\left\{A_{i}\right\}}_{i=1,2}\right)}≜2^{h_{N}^{\mathrm{ib}}}\;. \end{array}$$

Considering the exponential terms on the right-hand side (RHS) of the inequality in (A5), we have

(A6) $$\begin{array}{cc} \frac{1}{N}h\left(w_{i}|{\left\{A_{i}\right\}}_{i=1,2}\right) & =\frac{1}{N}\;E\left\{\log\left({\left(\pi e\right)}^{N}\det\left(\kappa_{i}I_{N}+\frac{{\mathrm{snr}}_{i}}{d}A_{i}A_{i}^{†}\right)\right)\right\}\\ \; & =\log\left(\pi e\right)+\log\kappa_{i}+\frac{1}{N}\;E\left\{\log\left(\det\left(I_{N}+\frac{{\mathrm{snr}}_{i}/\kappa_{i}}{d}A_{i}A_{i}^{†}\right)\right)\right\}\\ \; & \overset{\left(a\right)}{\underset{N\to \infty }{\to }}\log\left(\pi e\right)+\log\kappa_{i}+C_{i}\left(\frac{{\mathrm{snr}}_{i}}{\kappa_{i}}\right)\;,\;i=1,2\;, \end{array}$$

where (a) follows from Theorem 1 (note that the introduction of the parameter κ in the *open* interval (0,1), as part of the noise-split step (A2), is necessary for the applicability of Theorem 1). Then, considering the large-system limit and substituting (A6) back into (A5), while relying on the continuity of the exponential and logarithmic functions, we obtain the following lower bound:

(A7) $$\frac{1}{N}h\left(y|{\left\{A_{i}\right\}}_{i=1,2}\right)\geq h_{N}^{\mathrm{ib}}\underset{N\to \infty }{\to }\log\left(\pi e\right)+\log\left(\kappa_{1}2^{C_{1}\left(\frac{{\mathrm{snr}}_{1}}{\kappa_{1}}\right)}+\kappa_{2}2^{C_{2}\left(\frac{{\mathrm{snr}}_{2}}{\kappa_{2}}\right)}\right).$$

Further denoting

(A8) $$R_{N,\mathrm{sum}}^{\mathrm{ib}}≜h_{N}^{\mathrm{ib}}-\log\left(\pi e\right)\;,$$

we thus finally conclude that the normalized mutual information (A1) can be lower-bounded as

(A9) $$\frac{1}{N}I\left(x_{1},x_{2};y|{\left\{A_{i}\right\}}_{i=1,2}\right)\geq R_{N,\mathrm{sum}}^{\mathrm{ib}}\underset{N\to \infty }{\to }\log\left(\kappa_{1}2^{C_{1}\left(\frac{{\mathrm{snr}}_{1}}{\kappa_{1}}\right)}+\kappa_{2}2^{C_{2}\left(\frac{{\mathrm{snr}}_{2}}{\kappa_{2}}\right)}\right),$$

which, when combined with (9), yields (10). This completes the proof of the proposition.

## Appendix B. Proof of Proposition 2

Analogously to the proof of Proposition 1 in Appendix A, we aim to *upper*-bound the sum-rate constraint in (8). Towards this end, we employ a *strengthened* version of the EPI, recently derived in [45] (see also [44]), which is adapted here to the complex setting.

**Theorem** **A1** ([45], Corollary 2). *Suppose that x and w are random vectors in $C^{N}$, conditionally independent given Q; moreover, suppose that w is conditionally Gaussian given Q. Define y=x+w. For any v satisfying x→y→v|Q,*

(A10) $$2^{\frac{1}{N}\left(h\left(y|Q\right)-I\left(x;v|Q\right)\right)}\geq 2^{\frac{1}{N}\left(h\left(x|Q\right)-I\left(y;v|Q\right)\right)}+2^{\frac{1}{N}h\left(w|Q\right)}\;.$$

Theorem A1 immediately implies the following conditional EPI for *three* random summands (one of which is Gaussian).

**Corollary** **A1** ([45], Theorem 3). *Let x, y and w be three random vectors in $C^{N}$, conditionally independent given Q, with finite second moments. Moreover, conditional on Q, let w be distributed as CN(0,Σ). Then,*

(A11) $$2^{\frac{1}{N}\left(h\left(x+w|Q\right)+h\left(y+w|Q\right)\right)}\geq 2^{\frac{1}{N}\left(h\left(x|Q\right)+h\left(y|Q\right)\right)}+2^{\frac{1}{N}\left(h\left(x+y+w|Q\right)+h\left(w|Q\right)\right)}\;.$$

**Proof.** The Corollary follows from Theorem A1 by letting v=x+y+w and rearranging the exponents [45]. □

Now, let us fix $\mu_{1},\mu_{2}\in \left(0,1\right)$, such that

(A12) $$\mu_{3}≜1-\mu_{1}-\mu_{2}>0\;,$$

and let us consider the following three *independent* random vectors:

(A13) $$\begin{array}{ccc} \overset{ˇ}{x} & ≜ & \sqrt{\frac{{\mathrm{snr}}_{1}}{d}}A_{1}x_{1}+{\overset{ˇ}{z}}_{1}\;, \end{array}$$

(A14) $$\begin{array}{ccc} \overset{ˇ}{y} & ≜ & \sqrt{\frac{{\mathrm{snr}}_{2}}{d}}A_{2}x_{2}+{\overset{ˇ}{z}}_{2}\;, \end{array}$$

(A15) $$\begin{array}{ccc} \overset{ˇ}{w} & ≜ & {\overset{ˇ}{z}}_{3}\;, \end{array}$$

where ${\overset{ˇ}{z}}_{j}∼CN\left(0,\mu_{j}I_{N}\right)$, j=1,2,3, are independent complex Gaussian vectors, independent also of $x_{1}$, $x_{2}$ and ${\left\{A_{i}\right\}}_{i=1,2}$. Note that $\overset{ˇ}{x}$, $\overset{ˇ}{y}$ and $\overset{ˇ}{w}$ are independent conditional on the signature matrices ${\left\{A_{i}\right\}}_{i=1,2}$ as well. Then, observing that

(A16) $$y\overset{d}{=}\overset{ˇ}{x}+\overset{ˇ}{y}+\overset{ˇ}{w}$$

hence,

(A17) $$2^{\frac{1}{N}h\left(y|{\left\{A_{i}\right\}}_{i=1,2}\right)}=2^{\frac{1}{N}h\left(\overset{ˇ}{x}+\overset{ˇ}{y}+\overset{ˇ}{w}|{\left\{A_{i}\right\}}_{i=1,2}\right)}\;,$$

Theorem A1 and Corollary A1 can be applied to yield the upper bound

(A18) 21Nh(y|Aii=1,2)≤2hNub≜2−1Nh(wˇ)(21Nh(xˇ+wˇ|Aii=1,2)+1Nh(yˇ+wˇ|Aii=1,2)2hNub≜2−1Nh(wˇ)(21Nh(xˇ+wˇ|Aii=1,2)−21Nh(xˇ|Aii=1,2)+1Nh(yˇ|Aii=1,2)),

where ${\left\{A_{i}\right\}}_{i=1,2}$ take the role of *Q* and all differential entropies are neither *∞* nor −∞ by construction.

Starting with the Gaussian vector $\overset{ˇ}{w}$, its normalized differential entropy reads

(A19) $$\frac{1}{N}h\left(\overset{ˇ}{w}\right)=\log\left(\pi e\right)+\log\mu_{3}\;.$$

Next, considering each exponential term inside the parentheses in the RHS of (A18), we obtain, for the first term,

(A20) 1Nh(xˇ+wˇ|Aii=1,2)=1NElog(πe)Ndet(μ1+μ3)IN+snr1dA1A1†h(xˇ+wˇ)=(a)1NElog(πe)Ndetμ¯2IN+snr1dA1A1†h(xˇ+wˇ)=log(πe)+logμ¯2+1NElogdetIN+snr1/μ¯2dA1A1†h(xˇ+wˇ)→N→∞(b)log(πe)+logμ¯2+C1opt(snr1μ¯2),

where (a) is due to (A12) and (b) follows from Theorem 1. Treating the remaining terms in an analogous manner, we obtain

(A21) $$\begin{array}{ccc} \frac{1}{N}h\left(\overset{ˇ}{y}+\overset{ˇ}{w}|\left\{A_{i}\right\}\right) & \underset{N\to \infty }{\to } & \log\left(\pi e\right)+\log{\bar{\mu }}_{1}+C_{2}\left(\frac{{\mathrm{snr}}_{2}}{{\bar{\mu }}_{1}}\right)\;,\; \end{array}$$

(A22) $$\begin{array}{ccc} \frac{1}{N}h\left(\overset{ˇ}{x}\;|\left\{A_{i}\right\}\right) & \underset{N\to \infty }{\to } & \log\left(\pi e\right)+\log\mu_{1}+C_{1}\left(\frac{{\mathrm{snr}}_{1}}{\mu_{1}}\right)\;, \end{array}$$

(A23) $$\begin{array}{ccc} \frac{1}{N}h\left(\overset{ˇ}{y}\;|\left\{A_{i}\right\}\right) & \underset{N\to \infty }{\to } & \log\left(\pi e\right)+\log\mu_{2}+C_{2}\left(\frac{{\mathrm{snr}}_{2}}{\mu_{2}}\right)\;. \end{array}$$

Then, substituting (A19)–(A23) back into (A18), it follows, by continuity (with the aid of some algebra), that

(A24) $$\begin{array}{cc} \frac{1}{N}h\left(y|{\left\{A_{i}\right\}}_{i=1,2}\right)\leq h_{N}^{\mathrm{ub}}\; & \underset{N\to \infty }{\to }\log\left(\pi e\right)-\log\mu_{3}\\ \; & +\log\left({\bar{\mu }}_{1}{\bar{\mu }}_{2}\;2^{C_{1}\left(\frac{{\mathrm{snr}}_{1}}{{\bar{\mu }}_{2}}\right)+C_{2}\left(\frac{{\mathrm{snr}}_{2}}{{\bar{\mu }}_{1}}\right)}-\mu_{1}\mu_{2}\;2^{C_{1}\left(\frac{{\mathrm{snr}}_{1}}{\mu_{1}}\right)+C_{2}\left(\frac{{\mathrm{snr}}_{2}}{\mu_{2}}\right)}\right)\;. \end{array}$$

Finally, denoting

(A25) $$R_{N,\mathrm{sum}}^{\mathrm{ub}}≜h_{N}^{\mathrm{ub}}-\log\left(\pi e\right)\;,$$

we conclude that

(A26) $$\begin{array}{cc} \frac{1}{N} & I\left(x_{1},x_{2};y|{\left\{A_{i}\right\}}_{i=1,2}\right)\leq R_{N,\mathrm{sum}}^{\mathrm{ub}}\\ \; & \;\underset{N\to \infty }{\to }\log\left({\bar{\mu }}_{1}{\bar{\mu }}_{2}2^{C_{1}\left(\frac{{\mathrm{snr}}_{1}}{{\bar{\mu }}_{2}}\right)+C_{2}\left(\frac{{\mathrm{snr}}_{2}}{{\bar{\mu }}_{1}}\right)}-\mu_{1}\mu_{2}2^{C_{1}\left(\frac{{\mathrm{snr}}_{1}}{\mu_{1}}\right)+C_{2}\left(\frac{{\mathrm{snr}}_{2}}{\mu_{2}}\right)}\right)-\log\mu_{3}\;, \end{array}$$

which, together with (9), yields (12). This completes the proof of Proposition 2.

## Appendix C. Proof of Proposition 4

Let $C_{\mathrm{sum}}^{\mathrm{lb}}\left({\mathrm{snr}}_{\mathrm{av}}\right)$ denote the lower bound on the achievable sum rate (27), when expressed in *nats/channel use* per dimension as a function of ${\mathrm{snr}}_{\mathrm{av}}$. Then,

(A27) $$C_{\mathrm{sum}}^{\mathrm{lb}}\left({\mathrm{snr}}_{\mathrm{av}}\right)=\ln\left(\kappa_{1}e^{C_{1}\left(\frac{\chi {\mathrm{snr}}_{\mathrm{av}}}{\kappa_{1}}\right)}+\kappa_{2}e^{C_{2}\left(\frac{\tilde{\alpha }\chi {\mathrm{snr}}_{\mathrm{av}}}{\kappa_{2}}\right)}\right)\;,$$

where we use $C_{i}\left(\cdot \right)$ to denote the function $C_{i}\left(\cdot \right)$ converted to nats/channel use, i=1,2 (cf. (5)). The minimum $\frac{E_{b}}{N_{0}}$ that enables reliable communications and the low-SNR slope of the sum-rate lower bound then read (cf. (24)–(25))

(A28) $${\left(\frac{E_{b}}{N_{0}}\right)}_{\min}^{\mathrm{lb}}=\frac{\beta \ln2}{{\dot{C}}_{\mathrm{sum}}^{\mathrm{lb}}\left(0\right)}\;,\;S_{0}^{\mathrm{lb}}=-\frac{2{\left[{\dot{C}}_{\mathrm{sum}}^{\mathrm{lb}}\left(0\right)\right]}^{2}}{{\ddot{C}}_{\mathrm{sum}}^{\mathrm{lb}}\left(0\right)}\;.$$

Starting with the first derivative of (A27), we obtain

(A29) $${\dot{C}}_{\mathrm{sum}}^{\mathrm{lb}}\left({\mathrm{snr}}_{\mathrm{av}}\right)=\frac{\kappa_{1}e^{C_{1}\left(\frac{\chi {\mathrm{snr}}_{\mathrm{av}}}{\kappa_{1}}\right)}\frac{d}{d{\mathrm{snr}}_{\mathrm{av}}}C_{1}\left(\frac{\chi {\mathrm{snr}}_{\mathrm{av}}}{\kappa_{1}}\right)+\kappa_{2}e^{C_{2}\left(\frac{\tilde{\alpha }\chi {\mathrm{snr}}_{\mathrm{av}}}{\kappa_{2}}\right)}\frac{d}{d{\mathrm{snr}}_{\mathrm{av}}}C_{2}\left(\frac{\tilde{\alpha }\chi {\mathrm{snr}}_{\mathrm{av}}}{\kappa_{2}}\right)}{\kappa_{1}e^{C_{1}\left(\frac{\chi {\mathrm{snr}}_{\mathrm{av}}}{\kappa_{1}}\right)}+\kappa_{2}e^{C_{2}\left(\frac{\tilde{\alpha }\chi {\mathrm{snr}}_{\mathrm{av}}}{\kappa_{2}}\right)}}\;,\;$$

which, using the fact that $C_{i}\left(0\right)=0$ and recalling that $\kappa_{1}+\kappa_{2}=1$, yields

(A30) $${\dot{C}}_{\mathrm{sum}}^{\mathrm{lb}}\left(0\right)=\chi \left({\dot{C}}_{1}\left(0\right)+\tilde{\alpha }{\dot{C}}_{2}\left(0\right)\right)\;.$$

Now, as shown in [38,39] (see the proof of Theorem 3 therein), the asymptotic normalized spectral efficiency $C_{i}\left({\mathrm{snr}}_{i}\right)$, i=1,2 (cf. (5) and (9)), coincides with the Shannon transform of the asymptotic empirical eigenvalue distribution of $\frac{1}{d}A_{i}A_{i}^{†}$, whose density reads ([38], Theorem 2) (see also [39])

(A31) $$\rho \left(\lambda ,\beta_{i},d\right)={\left[1-\beta_{i}\right]}^{+}\delta \left(\lambda \right)+\frac{\beta_{i}d}{2\pi }\frac{\sqrt{{\left[\lambda -\lambda_{i}^{-}\right]}^{+}{\left[\lambda_{i}^{+}-\lambda \right]}^{+}}}{\lambda (\beta_{i}d-\lambda )}\;,$$

where $\lambda_{i}^{\pm }={\left(\sqrt{\alpha }\pm \sqrt{\gamma_{i}}\right)}^{2}$, i=1,2, δ(λ) is a unit point mass at λ=0 and ${\left[z\right]}^{+}≜\max\left\{0,z\right\}$. Let F be a probability distribution defined on $R^{+}$. The Shannon transform $V_{F}$ of F is defined for $x\in R^{+}$ as $V\left(x\right)≜\int_{R^{+}}\log\left(1+x\lambda \right)dF\left(\lambda \right)$ [30], Definition 3.2 (see also [29], Definition 2.12). Hence, the derivative at zero ${\dot{C}}_{i}\left(0\right)$, i=1,2, reads

(A32) $${\dot{C}}_{i}\left(0\right)=\frac{d}{d{\mathrm{snr}}_{\mathrm{av}}}\left(\frac{\beta_{i}d}{2\pi }\int_{\lambda_{i}^{-}}^{\lambda_{i}^{+}}\ln\left(1+{\mathrm{snr}}_{\mathrm{av}}\lambda \right)\frac{\sqrt{\left(\lambda -\lambda_{i}^{-}\right)\left(\lambda_{i}^{+}-\lambda \right)}}{\lambda (\beta_{i}d-\lambda )}\;d\lambda \right){|}_{{\mathrm{snr}}_{\mathrm{av}}=0}=\beta_{i}\;.\;$$

Thus, we conclude, from (A30) and (21), that

(A33) $${\dot{C}}_{\mathrm{sum}}^{\mathrm{lb}}\left(0\right)=\chi \left(\beta_{1}+\tilde{\alpha }\beta_{2}\right)=\beta \;.$$

Turning to the second derivative, we have

(A34) C¨sumlb(snrav)=ddsnravκ1eC1(χsnravκ1)ddsnravC1(χsnravκ1)κ1eC1(χsnravκ1)+κ2eC2(α˜χsnravκ2)=+ddsnravκ2eC2(α˜χsnravκ2)ddsnravC2(α˜χsnravκ2)κ1eC1(χsnravκ1)+κ2eC2(α˜χsnravκ2)=−κ1eC1(χsnravκ1)ddsnravC1(χsnravκ1)+κ2eC2(α˜χsnravκ2)ddsnravC2(α˜χsnravκ2)2κ1eC1(χsnravκ1)+κ2eC2(α˜χsnravκ2)2.

Since the denominators of all three terms in (A34) approach unity as ${\mathrm{snr}}_{\mathrm{av}}\to 0$, we focus on the numerators. Starting with the first term, note that

(A35) $$\begin{array}{cc} \frac{d}{d{\mathrm{snr}}_{\mathrm{av}}}\left(\kappa_{1}e^{C_{1}\left(\frac{\chi {\mathrm{snr}}_{\mathrm{av}}}{\kappa_{1}}\right)}\frac{d}{d{\mathrm{snr}}_{\mathrm{av}}}C_{1}\left(\frac{\chi {\mathrm{snr}}_{\mathrm{av}}}{\kappa_{1}}\right)\right)\; & \underset{{\mathrm{snr}}_{\mathrm{av}}\to 0}{\to }\kappa_{1}{\left(\frac{\chi }{\kappa_{1}}{\dot{C}}_{1}\left(0\right)\right)}^{2}+\kappa_{1}{\left(\frac{\chi }{\kappa_{1}}\right)}^{2}{\ddot{C}}_{1}\left(0\right)\\ \; & =\frac{\chi^{2}}{\kappa_{1}}\left({\left({\dot{C}}_{1}\left(0\right)\right)}^{2}+{\ddot{C}}_{1}\left(0\right)\right)\;. \end{array}$$

Next, similarly to (A32), we obtain, for i=1,2,

(A36) $$\begin{array}{cc} {\ddot{C}}_{i}\left(0\right) & =\frac{d^{2}}{d{\mathrm{snr}}_{\mathrm{av}}^{2}}\left(\frac{\beta_{i}d}{2\pi }\int_{\lambda_{i}^{-}}^{\lambda_{i}^{+}}\ln\left(1+{\mathrm{snr}}_{\mathrm{av}}\lambda \right)\frac{\sqrt{\left(\lambda -\lambda_{i}^{-}\right)\left(\lambda_{i}^{+}-\lambda \right)}}{\lambda (\beta_{i}d-\lambda )}\;d\lambda \right){|}_{{\mathrm{snr}}_{\mathrm{av}}=0}\\ \; & =-\frac{\beta_{i}\left(d\left(1+\beta_{i}\right)-1\right)}{d}\;, \end{array}$$

yielding

(A37) $$\begin{array}{cc} \frac{d}{d{\mathrm{snr}}_{\mathrm{av}}}\left(\kappa_{1}e^{C_{1}\left(\frac{\chi {\mathrm{snr}}_{\mathrm{av}}}{\kappa_{1}}\right)}\frac{d}{d{\mathrm{snr}}_{\mathrm{av}}}C_{1}\left(\frac{\chi {\mathrm{snr}}_{\mathrm{av}}}{\kappa_{1}}\right)\right)\; & \underset{{\mathrm{snr}}_{\mathrm{av}}\to 0}{\to }-\frac{\chi^{2}}{\kappa_{1}}\cdot \frac{\beta_{1}\left(d-1\right)}{d}\;. \end{array}$$

In a completely analogous manner, the numerator of the second term in (A34) takes the following form as ${\mathrm{snr}}_{\mathrm{av}}\to 0$:

(A38) $$\frac{d}{d{\mathrm{snr}}_{\mathrm{av}}}\left(\kappa_{2}e^{C_{2}\left(\frac{\alpha \chi {\mathrm{snr}}_{\mathrm{av}}}{\kappa_{2}}\right)}\frac{d}{d{\mathrm{snr}}_{\mathrm{av}}}C_{2}\left(\frac{\alpha \chi {\mathrm{snr}}_{\mathrm{av}}}{\kappa_{2}}\right)\right)\underset{{\mathrm{snr}}_{\mathrm{av}}\to 0}{\to }-\frac{{\tilde{\alpha }}^{2}\chi^{2}}{\kappa_{2}}\cdot \frac{\beta_{2}\left(d-1\right)}{d}\;.$$

Substituting (A37) and (A38) back into (A34), while taking the limit ${\mathrm{snr}}_{\mathrm{av}}\to 0$, we finally obtain with the aid of (A33) and (21),

(A39) $$\begin{array}{cc} {\ddot{C}}_{\mathrm{sum}}^{\mathrm{lb}}\left(0\right) & =-\frac{\chi^{2}}{\kappa_{1}}\cdot \frac{\beta_{1}\left(d-1\right)}{d}-\frac{{\tilde{\alpha }}^{2}\chi^{2}}{\kappa_{2}}\cdot \frac{\beta_{2}\left(d-1\right)}{d}-\beta^{2}\\ \; & =-\beta^{2}\left(1+\frac{d-1}{d{\left(\beta_{1}+\tilde{\alpha }\beta_{2}\right)}^{2}}\cdot \left(\frac{\beta_{1}}{\kappa_{1}}+\frac{{\tilde{\alpha }}^{2}\beta_{2}}{\kappa_{2}}\right)\right)\;. \end{array}$$

The proof of Proposition 4 is then completed by substituting (A33) and (A39) back into (A28).

## Appendix D. Proof of Proposition 5

Following similar steps to the proof in Appendix C, while retaining the notation therein, let $C_{\mathrm{sum}}^{\mathrm{ub}}\left({\mathrm{snr}}_{\mathrm{av}}\right)$ denote the sum-rate upper bound (28) in *nats/channel use* per dimension. Then,

(A40) $$\begin{array}{cc} C_{\mathrm{sum}}^{\mathrm{ub}}\left({\mathrm{snr}}_{\mathrm{av}}\right) & =\ln\left({\bar{\mu }}_{1}{\bar{\mu }}_{2}e^{C_{1}\left(\frac{\chi {\mathrm{snr}}_{\mathrm{av}}}{{\bar{\mu }}_{2}}\right)+C_{2}\left(\frac{\tilde{\alpha }\chi {\mathrm{snr}}_{\mathrm{av}}}{{\bar{\mu }}_{1}}\right)}-\mu_{1}\mu_{2}e^{C_{1}\left(\frac{\chi {\mathrm{snr}}_{\mathrm{av}}}{\mu_{1}}\right)+C_{2}\left(\frac{\tilde{\alpha }\chi {\mathrm{snr}}_{\mathrm{av}}}{\mu_{2}}\right)}\right)-\ln{\bar{\mu }}_{12}\;. \end{array}$$

It is important to note, at this point, that, in contrast to the sum-rate lower bound (27), the sum-rate upper bound (A40) may exhibit a bowl-shaped form when plotted as a function of $\frac{E_{b}}{N_{0}}$ in dB (see, e.g., the discussion in ([56], p. 1341)). This behavior depends on the system load parameters and the particular choice of the constants $\mu_{1}$ and $\mu_{2}$. That said, insights on the low-SNR characteristics of this upper bound can still be obtained by deriving its low-SNR parameters. One should only bear in mind that the value of $\frac{E_{b}}{N_{0}}$ corresponding to ${\mathrm{snr}}_{\mathrm{av}}\to 0$ *does not* necessarily represent the minimum $\frac{E_{b}}{N_{0}}$ above which the upper bound is positive. In fact, the latter may be attained at a *lower*$\frac{E_{b}}{N_{0}}$, as discussed in [56]. In such case, the low-SNR slope turns out to be negative, as shall be made clear in the sequel.

The low-SNR parameters of $C_{\mathrm{sum}}^{\mathrm{ub}}\left({\mathrm{snr}}_{\mathrm{av}}\right)$ are given by

(A41) $${\left(\frac{E_{b}}{N_{0}}\right)}_{0}^{\mathrm{ub}}=\frac{\beta \ln2}{{\dot{C}}_{\mathrm{sum}}^{\mathrm{ub}}\left(0\right)}\;,$$

(A42) $$S_{0}^{\mathrm{ub}}=-\frac{2{\left[{\dot{C}}_{\mathrm{sum}}^{\mathrm{ub}}\left(0\right)\right]}^{2}}{{\ddot{C}}_{\mathrm{sum}}^{\mathrm{ub}}\left(0\right)}\;,$$

with the subscript ${\left\{\cdot \right\}}_{0}$ in (A41) designating $\frac{E_{b}}{N_{0}}$ that corresponds to ${\mathrm{snr}}_{\mathrm{av}}\to 0$. Hence, we proceed by taking the first derivative of $C_{\mathrm{sum}}^{\mathrm{ub}}\left({\mathrm{snr}}_{\mathrm{av}}\right)$,

(A43) C˙sumub(snrav)=μ¯1μ¯2eC1(χsnravμ¯2)+C2(α˜χsnravμ¯1)ddsnravC1(χsnravμ¯2)+ddsnravC2(α˜χsnravμ¯1)μ¯1μ¯2eC1(χsnravμ¯2)+C2(α˜χsnravμ¯1)−μ1μ2eC1(χsnravμ1)+C2(α˜χsnravμ2)=−μ1μ2eC1(χsnravμ1)+C2(α˜χsnravμ2)ddsnravC1(χsnravμ1)+ddsnravC2(α˜χsnravμ2)μ¯1μ¯2eC1(χsnravμ¯2)+C2(α˜χsnravμ¯1)−μ1μ2eC1(χsnravμ1)+C2(α˜χsnravμ2),

which, letting ${\mathrm{snr}}_{\mathrm{av}}\to 0$ and using (A32), yields

(A44) $$\begin{array}{cc} {\dot{C}}_{\mathrm{sum}}^{\mathrm{ub}}\left(0\right) & =\frac{{\bar{\mu }}_{1}{\bar{\mu }}_{2}\left(\frac{\chi }{{\bar{\mu }}_{2}}{\dot{C}}_{1}\left(0\right)+\frac{\tilde{\alpha }\chi }{{\bar{\mu }}_{1}}{\dot{C}}_{2}\left(0\right)\right)-\mu_{1}\mu_{2}\left(\frac{\chi }{\mu_{1}}{\dot{C}}_{1}\left(0\right)+\frac{\tilde{\alpha }\chi }{\mu_{2}}{\dot{C}}_{2}\left(0\right)\right)}{{\bar{\mu }}_{1}{\bar{\mu }}_{2}-\mu_{1}\mu_{2}}=\chi \left(\beta_{1}+\tilde{\alpha }\beta_{2}\right)=\beta \;. \end{array}$$

Therefore, it is concluded, from (A41), that, as ${\mathrm{snr}}_{\mathrm{av}}\to 0$ (implying $C_{\mathrm{sum}}^{\mathrm{ub}}\left({\mathrm{snr}}_{\mathrm{av}}\right)\to 0$), the corresponding $\frac{E_{b}}{N_{0}}$ approaches

(A45) $${\left(\frac{E_{b}}{N_{0}}\right)}_{0}^{\mathrm{ub}}=\ln2\;,$$

which, as said, does not necessarily correspond to the minimum $\frac{E_{b}}{N_{0}}$ above which the sum-rate upper bound is positive.

To derive the slope of the sum-rate upper bound at ${\mathrm{snr}}_{\mathrm{av}}\to 0$, we take the second derivative of $C_{\mathrm{sum}}^{\mathrm{ub}}\left({\mathrm{snr}}_{\mathrm{av}}\right)$. From (A43), we obtain

(A46) C¨sumub(snrav)=C¨sumubddsnravμ¯1μ¯2eC1(χsnravμ¯2)+C2(α˜χsnravμ¯1)ddsnravC1(χsnravμ¯2)+ddsnravC2(α˜χsnravμ¯1)μ¯1μ¯2eC1(χsnravμ¯2)+C2(α˜χsnravμ¯1)−μ1μ2eC1(χsnravμ1)+C2(α˜χsnravμ2)C¨sumub−ddsnravμ1μ2eC1(χsnravμ1)+C2(α˜χsnravμ2)ddsnravC1(χsnravμ1)+ddsnravC2(α˜χsnravμ2)μ¯1μ¯2eC1(χsnravμ¯2)+C2(α˜χsnravμ¯1)−μ1μ2eC1(χsnravμ1)+C2(α˜χsnravμ2)C¨sumub−μ¯1μ¯2eC1(χsnravμ¯2)+C2(α˜χsnravμ¯1)ddsnravC1(χsnravμ¯2)+ddsnravC2(α˜χsnravμ¯1)C¨sumub−μ1μ2eC1(χsnravμ1)+C2(α˜χsnravμ2)ddsnravC1(χsnravμ1)+ddsnravC2(α˜χsnravμ2)2C¨sumub·μ¯1μ¯2eC1(χsnravμ¯2)+C2(α˜χsnravμ¯1)−μ1μ2eC1(χsnravμ1)+C2(α˜χsnravμ2)−2.

In view of (A44), the last term in (A46) converges to $\beta^{2}$ as ${\mathrm{snr}}_{\mathrm{av}}\to 0$, while the denominators of the first two terms converge to ${\bar{\mu }}_{12}$. Therefore, it is left to calculate the limit of the respective numerators at ${\mathrm{snr}}_{\mathrm{av}}\to 0$. Starting with the first term in (A46), we have

(A47) ddsnravμ¯1μ¯2eC1(χsnravμ¯2)+C2(α˜χsnravμ¯1)ddsnravC1(χsnravμ¯2)+ddsnravC2(α˜χsnravμ¯1)ddsnrav=→snrav→0Aub,

where

(A48) $$\begin{array}{cc} A^{\mathrm{ub}}\; & =\frac{{\bar{\mu }}_{1}\chi^{2}}{{\bar{\mu }}_{2}}\left({\left({\dot{C}}_{1}\left(0\right)\right)}^{2}+{\ddot{C}}_{1}\left(0\right)\right)+\frac{{\bar{\mu }}_{2}{\tilde{\alpha }}^{2}\chi^{2}}{{\bar{\mu }}_{1}}\left({\left({\dot{C}}_{2}\left(0\right)\right)}^{2}+{\ddot{C}}_{2}\left(0\right)\right)+2\tilde{\alpha }\chi^{2}{\dot{C}}_{1}\left(0\right){\dot{C}}_{2}\left(0\right)\;\;. \end{array}$$

Relying on (A32) and (A36), we then finally obtain (following some algebra)

(A49) $$A^{\mathrm{ub}}=-\frac{{\bar{\mu }}_{1}\chi^{2}}{{\bar{\mu }}_{2}}\frac{\beta_{1}\left(d-1\right)}{d}-\frac{{\bar{\mu }}_{2}{\tilde{\alpha }}^{2}\chi^{2}}{{\bar{\mu }}_{1}}\frac{\beta_{2}\left(d-1\right)}{d}+2\tilde{\alpha }\chi^{2}\beta_{1}\beta_{2}\;.$$

Similarly, the numerator of the second term in (A46) reads

(A50) ddsnravμ1μ2eC1(χsnravμ1)+C2(α˜χsnravμ2)ddsnravC1(χsnravμ1)+ddsnravC2(α˜χsnravμ2)ddsnrav=→snrav→0Bub,

where

(A51) $$\begin{array}{cc} B^{\mathrm{ub}}\; & =\frac{\mu_{2}\chi^{2}}{\mu_{1}}\left({\left({\dot{C}}_{1}\left(0\right)\right)}^{2}+{\ddot{C}}_{1}\left(0\right)\right)+\frac{\mu_{1}{\tilde{\alpha }}^{2}\chi^{2}}{\mu_{2}}\left({\left({\dot{C}}_{2}\left(0\right)\right)}^{2}+{\ddot{C}}_{2}\left(0\right)\right)+2\tilde{\alpha }\chi^{2}{\dot{C}}_{1}\left(0\right){\dot{C}}_{2}\left(0\right)\;\;. \end{array}$$

Similarly to (A49), this yields

(A52) $$B^{\mathrm{ub}}=-\frac{\mu_{2}\chi^{2}}{\mu_{1}}\frac{\beta_{1}\left(d-1\right)}{d}-\frac{\mu_{1}{\tilde{\alpha }}^{2}\chi^{2}}{\mu_{2}}\frac{\beta_{2}\left(d-1\right)}{d}+2\tilde{\alpha }\chi^{2}\beta_{1}\beta_{2}\;.$$

Finally, substituting (A49) and (A52) into (A46) while taking ${\mathrm{snr}}_{\mathrm{av}}\to 0$, we obtain

(A53) $$\begin{array}{cc} {\ddot{C}}_{\mathrm{sum}}^{\mathrm{ub}}\left(0\right) & =\frac{A^{\mathrm{ub}}-B^{\mathrm{ub}}}{{\bar{\mu }}_{12}}-\beta^{2}\\ \; & =-\beta^{2}\left(1+\frac{\left(\mu_{1}-\mu_{2}\right)\left(d-1\right)}{d{\left(\beta_{1}+\tilde{\alpha }\beta_{2}\right)}^{2}}\left(\frac{\beta_{1}}{\mu_{1}{\bar{\mu }}_{2}}-\frac{{\tilde{\alpha }}^{2}\beta_{2}}{\mu_{2}{\bar{\mu }}_{1}}\right)\right)\;. \end{array}$$

Hence, we conclude, from (A42), that the low-SNR slope is given by

(A54) $$\begin{array}{cc} S_{0}^{\mathrm{ub}}\; & =\frac{2}{1+\frac{\left(\mu_{1}-\mu_{2}\right)\left(d-1\right)}{d{\left(\beta_{1}+\tilde{\alpha }\beta_{2}\right)}^{2}}\left(\frac{\beta_{1}}{\mu_{1}{\bar{\mu }}_{2}}-\frac{{\tilde{\alpha }}^{2}\beta_{2}}{\mu_{2}{\bar{\mu }}_{1}}\right)}\;. \end{array}$$

Note that setting $\mu_{1}=\mu_{2}$ yields $S_{0}^{\mathrm{ub}}=2$, which is the low-SNR slope of the single-user AWGN channel, as well as the Cover–Wyner sum capacity. Furthermore, let D denote the set

(A55) $$\begin{array}{c} D≜\left\{\mu_{1},\mu_{2}:\mu_{1},\mu_{2}\in \left(0,1\right)\;,\;1-\mu_{1}-\mu_{2}>0\;,\;\left(\mu_{1}-\mu_{2}\right)\left(\frac{\beta_{1}}{\mu_{1}{\bar{\mu }}_{2}}-\frac{{\tilde{\alpha }}^{2}\beta_{2}}{\mu_{2}{\bar{\mu }}_{1}}\right)>0\right\}\;. \end{array}$$

Then, recalling that d≥2 (see Section 2), the low-SNR slope (A54) is *positive* and lower than 2 as long as $(\mu_{1},\mu_{2})\in D$. Hence, for the sum-rate upper bound (28) to be useful, the parameters $\mu_{1},\mu_{2}$ should be restricted in the low-SNR regime only to the set D. The fact that the low-SNR slope is positive over D immediately implies that (A45) is indeed the minimum $\frac{E_{b}}{N_{0}}$ over which the upper bound is positive in this setting. This completes the proof of Proposition 5. We further note that, for $(\mu_{1}\mu_{2})\notin D$, the low-SNR slope may turn out *negative* and it may exhibit discontinuity if $\mu_{1}$ and $\mu_{2}$ are chosen so that

(A56) $$1+\frac{\left(\mu_{1}-\mu_{2}\right)\left(d-1\right)}{d{\left(\beta_{1}+\tilde{\alpha }\beta_{2}\right)}^{2}}\left(\frac{\beta_{1}}{\mu_{1}{\bar{\mu }}_{2}}-\frac{{\tilde{\alpha }}^{2}\beta_{2}}{\mu_{2}{\bar{\mu }}_{1}}\right)=0\;.$$

The bound is obviously not useful in this regime.

## Appendix E. Proof of Proposition 6

Let $R({\mathrm{snr}}_{\mathrm{av}})$ denote an achievable rate in bit/sec/Hz expressed as a function of ${\mathrm{snr}}_{\mathrm{av}}$. Let us recall that the high-SNR approximation of $R({\mathrm{snr}}_{\mathrm{av}})$ reads

(A57) $$R\left({\mathrm{snr}}_{\mathrm{av}}\right)\approx S_{\infty }\left(\log{\mathrm{snr}}_{\mathrm{av}}-L_{\infty }\right)\;,\;{\mathrm{snr}}_{\mathrm{av}}\gg 1\;,$$

where $S_{\infty }$ denotes the high-SNR slope and $L_{\infty }$ denotes the high-SNR power offset. Then, for any constant a>0, $R(a\cdot {\mathrm{snr}}_{\mathrm{av}})$ can be characterized for ${\mathrm{snr}}_{\mathrm{av}}\gg 1$ as

(A58) $$R\left(a\cdot {\mathrm{snr}}_{\mathrm{av}}\right)\approx S_{\infty }\left(\log\left(a\cdot {\mathrm{snr}}_{\mathrm{av}}\right)-L_{\infty }\right)=S_{\infty }\left(\log{\mathrm{snr}}_{\mathrm{av}}-\left(L_{\infty }-\log a\right)\right)\;.$$

Hence, any scaling of ${\mathrm{snr}}_{\mathrm{av}}$ by a factor a>0 corresponds to a shift in the high-SNR power offset by (−loga).

Rewriting the lower bound on the achievable sum rate (27), we obtain

(A59) $$\begin{array}{cc} C_{\mathrm{sum}}^{\mathrm{lb}}\left({\mathrm{snr}}_{\mathrm{av}}\right)\; & =C_{2}\left(\frac{\tilde{\alpha }\chi {\mathrm{snr}}_{\mathrm{av}}}{\bar{\kappa }}\right)+\log\left(1+\kappa \left(2^{C_{1}\left(\frac{\chi {\mathrm{snr}}_{\mathrm{av}}}{\kappa }\right)-C_{2}\left(\frac{\tilde{\alpha }\chi {\mathrm{snr}}_{\mathrm{av}}}{\bar{\kappa }}\right)}-1\right)\right),\; \end{array}$$

where we use the identities $\kappa_{1}=\kappa$ and $\kappa_{2}=\bar{\kappa }=1-\kappa$. Let $S_{\infty ,i}$ and $L_{\infty ,i}$, i=1,2, denote the high-SNR parameters characterizing $C_{i}\left(\cdot \right)$ as in ([38], Proposition 5; see also [39], Proposition 4). Specifically, for i=1,2,

(A60) $$S_{\infty ,i}=\min\left(\beta_{i},1\right)\;,$$

and $L_{\infty ,i}$ is given in (62). Then, the term outside the logarithm in (A59) can be approximated for ${\mathrm{snr}}_{\mathrm{av}}\to \infty$ as

(A61) $$C_{2}\left(\frac{\tilde{\alpha }\chi {\mathrm{snr}}_{\mathrm{av}}}{\bar{\kappa }}\right)=S_{\infty ,2}\left(\log{\mathrm{snr}}_{\mathrm{av}}-\left(L_{\infty ,2}-\log\left(\frac{\tilde{\alpha }\chi }{\bar{\kappa }}\right)\right)\right)+o\left(1\right)\;.$$

Therefore, it is left to examine the different term in the exponent inside the logarithm in (A59), which we denote as

(A62) $$\Delta \left({\mathrm{snr}}_{\mathrm{av}}\right)≜C_{1}\left(\frac{\chi {\mathrm{snr}}_{\mathrm{av}}}{\kappa }\right)-C_{2}\left(\frac{\tilde{\alpha }\chi {\mathrm{snr}}_{\mathrm{av}}}{\bar{\kappa }}\right)\;.$$

Note that, accordingly, the sum-rate lower-bound (A59) can be compactly rewritten as

(A63) $$C_{\mathrm{sum}}^{\mathrm{lb}}\left({\mathrm{snr}}_{\mathrm{av}}\right)=C_{2}\left(\frac{\tilde{\alpha }\chi {\mathrm{snr}}_{\mathrm{av}}}{\bar{\kappa }}\right)+\log\left(1+\kappa \left(2^{\Delta ({\mathrm{snr}}_{\mathrm{av}})}-1\right)\right)\;.$$

We thus proceed with the analysis of (A63), while distinguishing among three possible cases and relying on the observations made in Appendix H.

### Appendix E.1. Case I: β 1,β 2 >1

Using (A133), we obtain, for ${\mathrm{snr}}_{\mathrm{av}}\to \infty$,

(A64) $$\begin{array}{cc} C_{1}\left(\frac{\chi {\mathrm{snr}}_{\mathrm{av}}}{\kappa }\right) & =\log{\mathrm{snr}}_{\mathrm{av}}+\log\left(\frac{\chi }{\kappa }\right)-L_{\infty ,1}+\frac{\kappa (\beta_{1}d-1)\log e}{\beta_{1}d\left(\beta_{1}-1\right)\chi }\;\frac{1}{{\mathrm{snr}}_{\mathrm{av}}}+o\left(\frac{1}{{\mathrm{snr}}_{\mathrm{av}}}\right)\;, \end{array}$$

where $L_{\infty ,1}$ should be interpreted here as the corresponding expression in (62) for $\beta_{1}>1$. Similarly, we obtain

(A65) $$\begin{array}{cc} C_{2}\left(\frac{\tilde{\alpha }\chi {\mathrm{snr}}_{\mathrm{av}}}{\bar{\kappa }}\right) & =\log{\mathrm{snr}}_{\mathrm{av}}+\log\left(\frac{\tilde{\alpha }\chi }{\bar{\kappa }}\right)-L_{\infty ,2}+\frac{\bar{\kappa }\left(\beta_{2}d-1\right)\log e}{\beta_{2}d\left(\beta_{2}-1\right)\tilde{\alpha }\chi }\;\frac{1}{{\mathrm{snr}}_{\mathrm{av}}}+o\left(\frac{1}{{\mathrm{snr}}_{\mathrm{av}}}\right)\;. \end{array}$$

The substitution back into (A62) then yields

(A66) $$\begin{array}{cc} \Delta ({\mathrm{snr}}_{\mathrm{av}}) & =\log\left(\frac{\bar{\kappa }}{\tilde{\alpha }\kappa }\right)+L_{\infty ,2}-L_{\infty ,1}+C_{1}\log e\cdot \frac{1}{{\mathrm{snr}}_{\mathrm{av}}}+o\left(\frac{1}{{\mathrm{snr}}_{\mathrm{av}}}\right)\;,\; \end{array}$$

where

(A67) $$C_{1}≜\left(\frac{\kappa (\beta_{1}d-1)}{\beta_{1}d\left(\beta_{1}-1\right)}-\frac{\bar{\kappa }\left(\beta_{2}d-1\right)}{\beta_{2}d\left(\beta_{2}-1\right)\tilde{\alpha }}\right)\cdot \frac{1}{\chi }\;.$$

Hence, for ${\mathrm{snr}}_{\mathrm{av}}\to \infty$, it can be shown (following some algebra) that

(A68) $$\begin{array}{cc} 2^{\Delta ({\mathrm{snr}}_{\mathrm{av}})}\; & =\frac{\bar{\kappa }}{\tilde{\alpha }\kappa }2^{L_{\infty ,2}-L_{\infty ,1}}\left(1+C_{1}\frac{1}{{\mathrm{snr}}_{\mathrm{av}}}+o\left(\frac{1}{{\mathrm{snr}}_{\mathrm{av}}}\right)\right)\;, \end{array}$$

(A69) $$\begin{array}{cc} \log\left(1+\kappa \left(2^{\Delta ({\mathrm{snr}}_{\mathrm{av}})}-1\right)\right)\; & =\log k_{1}+\frac{\bar{\kappa }}{\tilde{\alpha }k_{1}}2^{L_{\infty ,2}-L_{\infty ,1}}C_{1}\log e\cdot \frac{1}{{\mathrm{snr}}_{\mathrm{av}}}+o\left(\frac{1}{{\mathrm{snr}}_{\mathrm{av}}}\right)\;,\; \end{array}$$

where

(A70) $$k_{1}≜1+\frac{\bar{\kappa }}{\tilde{\alpha }}2^{L_{\infty ,2}-L_{\infty ,1}}-\kappa =\bar{\kappa }\left(1+\frac{1}{\tilde{\alpha }}2^{L_{\infty ,2}-L_{\infty ,1}}\right)\;,$$

and, finally (cf. (A63)),

(A71) $$C_{\mathrm{sum}}^{\mathrm{lb}}\left({\mathrm{snr}}_{\mathrm{av}}\right)=\log{\mathrm{snr}}_{\mathrm{av}}+\log\left(\frac{\tilde{\alpha }\chi }{\bar{\kappa }}\right)-L_{\infty ,2}+\log k_{1}+o\left(1\right)\;.$$

This lets us conclude that the high-SNR slope of the sum-rate lower bound reads

(A72) $$S_{\infty }^{\mathrm{lb}}=1\;,$$

and the respective high-SNR power offset satisfies

(A73) $$\begin{array}{cc} L_{\infty }^{\mathrm{lb}} & =L_{\infty ,2}-\log\left(\frac{\tilde{\alpha }\chi }{\bar{\kappa }}\right)-\log k_{1}\;. \end{array}$$

Using (A70), we finally conclude that

(A74) $$\begin{array}{cc} L_{\infty }^{\mathrm{lb}} & =L_{\infty ,1}+L_{\infty ,2}-\log\left(2^{L_{\infty ,1}}+\frac{1}{\tilde{\alpha }}2^{L_{\infty ,2}}\right)-\log\left(\tilde{\alpha }\chi \right)\;. \end{array}$$

### Appendix E.2. Case II: β 1 >1,β 2 =1

Using (A126), we obtain, for ${\mathrm{snr}}_{\mathrm{av}}\to \infty$,

(A75) $$\begin{array}{cc} C_{2}\left(\frac{\tilde{\alpha }\chi {\mathrm{snr}}_{\mathrm{av}}}{\bar{\kappa }}\right) & =\log{\mathrm{snr}}_{\mathrm{av}}+\log\frac{\tilde{\alpha }\chi }{\bar{\kappa }}-L_{\infty ,2}+2\sqrt{\frac{\left(d-1\right)\bar{\kappa }}{d\tilde{\alpha }\chi }}\log e\cdot \frac{1}{\sqrt{{\mathrm{snr}}_{\mathrm{av}}}}+o\left(\frac{1}{\sqrt{{\mathrm{snr}}_{\mathrm{av}}}}\right)\;. \end{array}$$

Combining (A75) with (A64) and substituting back into (A62), we obtain (while considering only small terms of leading order)

(A76) $$\begin{array}{cc} \Delta ({\mathrm{snr}}_{\mathrm{av}}) & =\log\left(\frac{\bar{\kappa }}{\tilde{\alpha }\kappa }\right)+L_{\infty ,2}-L_{\infty ,1}+C_{2}\log e\cdot \frac{1}{\sqrt{{\mathrm{snr}}_{\mathrm{av}}}}+o\left(\frac{1}{\sqrt{{\mathrm{snr}}_{\mathrm{av}}}}\right)\;, \end{array}$$

where

(A77) $$C_{2}≜-2\sqrt{\frac{\left(d-1\right)\bar{\kappa }}{d\tilde{\alpha }\chi }}\;.$$

This yields, for ${\mathrm{snr}}_{\mathrm{av}}\to \infty$,

(A78) $$\begin{array}{cc} 2^{\Delta ({\mathrm{snr}}_{\mathrm{av}})} & =\frac{\bar{\kappa }}{\tilde{\alpha }\kappa }2^{L_{\infty ,2}-L_{\infty ,1}}\left(1+C_{2}\frac{1}{\sqrt{{\mathrm{snr}}_{\mathrm{av}}}}+o(\frac{1}{\sqrt{{\mathrm{snr}}_{\mathrm{av}}})}\right)\;, \end{array}$$

(A79) $$\begin{array}{cc} \log\left(1+\kappa \left(2^{\Delta ({\mathrm{snr}}_{\mathrm{av}})}-1\right)\right) & =\log k_{2}+\frac{\bar{\kappa }}{\tilde{\alpha }k_{2}}2^{L_{\infty ,2}-L_{\infty ,1}}C_{2}\log e\cdot \frac{1}{\sqrt{{\mathrm{snr}}_{\mathrm{av}}}}+o\left(\frac{1}{\sqrt{{\mathrm{snr}}_{\mathrm{av}}}}\right)\;,\; \end{array}$$

where

(A80) $$k_{2}≜\bar{\kappa }\left(1+\frac{1}{\tilde{\alpha }}2^{L_{\infty ,2}-L_{\infty ,1}}\right)\;,$$

and, finally,

(A81) $$C_{\mathrm{sum}}^{\mathrm{lb}}\left({\mathrm{snr}}_{\mathrm{av}}\right)=\log{\mathrm{snr}}_{\mathrm{av}}+\log\left(\frac{\tilde{\alpha }\chi }{\bar{\kappa }}\right)-L_{\infty ,2}+\log k_{2}+o\left(1\right)\;.$$

Note that, although $k_{2}$ apparently takes the same form as $k_{1}$ in (A70), these two constants are not identical, since $L_{\infty ,2}$ differs for $\beta_{2}=1$ (cf. (62)); to emphasize this, we use a different notation.

Therefore, we conclude that the high-SNR slope of the sum-rate lower bound satisfies

(A82) $$S_{\infty }^{\mathrm{lb}}=1\;,$$

and the high-SNR power offset is given by

(A83) $$\begin{array}{cc} L_{\infty }^{\mathrm{lb}} & =L_{\infty ,2}-\log\left(\frac{\tilde{\alpha }\chi }{\bar{\kappa }}\right)-\log k_{2}\;. \end{array}$$

Using (A80), we then obtain

(A84) $$\begin{array}{cc} L_{\infty }^{\mathrm{lb}} & =L_{\infty ,1}^{\mathrm{opt}}+L_{\infty ,2}^{\mathrm{opt}}-\log\left(2^{L_{\infty ,1}}+\frac{1}{\tilde{\alpha }}2^{L_{\infty ,2}}\right)-\log\left(\tilde{\alpha }\chi \right)\;. \end{array}$$

### Appendix E.3. Case III: β 1 >1,2 d≤β 2 <1

For $\frac{2}{d}\leq \beta_{2}<1$, we start from (A140) and obtain, for ${\mathrm{snr}}_{\mathrm{av}}\to \infty$,

(A85) $$\begin{array}{cc} C_{2}^{\mathrm{opt}}\left(\frac{\tilde{\alpha }\chi {\mathrm{snr}}_{\mathrm{av}}}{\bar{\kappa }}\right) & =\beta_{2}\log{\mathrm{snr}}_{\mathrm{av}}+\beta_{2}\log\left(\frac{\tilde{\alpha }\chi }{\bar{\kappa }}\right)-\beta_{2}L_{\infty ,2}+\frac{\bar{\kappa }\beta_{2}^{3}\left(d-1\right)\log e}{d\left(1-\beta_{2}\right)\tilde{\alpha }\chi }\;\frac{1}{{\mathrm{snr}}_{\mathrm{av}}}+o\left(\frac{1}{{\mathrm{snr}}_{\mathrm{av}}}\right)\;. \end{array}$$

Using (A64) and substituting back into (A62), we then obtain

(A86) Δ(snrav)=(1−β2)logsnrav+logκ¯β2χ1−β2α˜β2κ+β2L∞,2−L∞,1=+C3loge·1snrav+o1snrav,

where

(A87) $$C_{3}≜\left(\frac{\kappa (\beta_{1}d-1)}{\beta_{1}d\left(\beta_{1}-1\right)}-\frac{\bar{\kappa }\beta_{2}^{3}\left(d-1\right)}{d\left(1-\beta_{2}\right)\tilde{\alpha }}\right)\frac{1}{\chi }\;.$$

This yields

(A88) $$\begin{array}{cc} 2^{\Delta ({\mathrm{snr}}_{\mathrm{av}})} & ={\mathrm{snr}}_{\mathrm{av}}^{1-\beta_{2}}\left(\frac{{\bar{\kappa }}^{\beta_{2}}\chi^{1-\beta_{2}}}{{\tilde{\alpha }}^{\beta_{2}}\kappa }\right)2^{\beta_{2}L_{\infty ,2}-L_{\infty ,1}}\left(1+C_{3}\frac{1}{{\mathrm{snr}}_{\mathrm{av}}}+o\left(\frac{1}{{\mathrm{snr}}_{\mathrm{av}}}\right)\right)\;, \end{array}$$

(A89) log1+κ2Δ(snrav)−1log2=(1−β2)logsnrav+β2L∞,2−L∞,1+logχ1−β2κ¯β2α˜β2+o(1),

hence,

(A90) $$\begin{array}{cc} C_{\mathrm{sum}}^{\mathrm{lb}}\left({\mathrm{snr}}_{\mathrm{av}}\right) & =\log{\mathrm{snr}}_{\mathrm{av}}-L_{\infty ,1}+\log_{2}\chi +o\left(1\right)\;. \end{array}$$

We finally conclude that the high-SNR slope for $\beta_{1}>1$ and $\frac{2}{d}\leq \beta_{2}<1$ reads

(A91) $$S_{\infty }^{\mathrm{lb}}=1\;,$$

while the high-SNR power offset reads

(A92) $$\begin{array}{cc} L_{\infty }^{\mathrm{lb}}\; & =L_{\infty ,1}-\log\chi \;. \end{array}$$

## Appendix F. Proof of Proposition 7

Starting from (28), we obtain, following some algebra,

(A93) Csumub(snrav)=C1(χsnravμ¯2)+C2(α˜χsnravμ¯1)=+log1−μ1μ2μ¯122C1(χsnravμ1)−C1(χsnravμ¯2)+C2(α˜χsnravμ2)−C2(α˜χsnravμ¯1)−1.

Next, relying on (A58), (A60) and (62), we conclude that the terms outside the logarithm in (A93) can be approximated, for ${\mathrm{snr}}_{\mathrm{av}}\to \infty$, as

(A94) C1(χsnravμ¯2)+C2(α˜χsnravμ¯1)=S∞,1+S∞,2logsnrav=−S∞,1L∞,1−logχ−S∞,2L∞,2−log(α˜χ)=−S∞,1log2μ¯2−S∞,2log2μ¯1+o(1).

Our next step is to investigate the different terms in the exponent inside the logarithm in (A93), which we compactly denote as

(A95) $$\begin{array}{ccc} \Delta_{1}\left({\mathrm{snr}}_{\mathrm{av}}\right) & ≜ & C_{1}\left(\frac{\chi {\mathrm{snr}}_{\mathrm{av}}}{\mu_{1}}\right)-C_{1}\left(\frac{\chi {\mathrm{snr}}_{\mathrm{av}}}{{\bar{\mu }}_{2}}\right)\;, \end{array}$$

(A96) $$\begin{array}{ccc} \Delta_{2}\left({\mathrm{snr}}_{\mathrm{av}}\right) & ≜ & C_{2}\left(\frac{\tilde{\alpha }\chi {\mathrm{snr}}_{\mathrm{av}}}{\mu_{2}}\right)-C_{2}\left(\frac{\tilde{\alpha }\chi {\mathrm{snr}}_{\mathrm{av}}}{{\bar{\mu }}_{1}}\right)\;. \end{array}$$

Focusing on $\beta_{1},\beta_{2}>1$, we resort to Appendix H and obtain, from (A133), that, for ${\mathrm{snr}}_{\mathrm{av}}\to \infty$,

(A97) $$\begin{array}{ccc} \Delta_{1}\left({\mathrm{snr}}_{\mathrm{av}}\right){|}_{\beta_{1}>1} & = & \log\left(\frac{{\bar{\mu }}_{2}}{\mu_{1}}\right)+\frac{\left(\mu_{1}-{\bar{\mu }}_{2}\right)\left(\beta_{1}d-1\right)\log e}{\beta_{1}d\left(\beta_{1}-1\right)\chi }\frac{1}{{\mathrm{snr}}_{\mathrm{av}}}+o\left(\frac{1}{{\mathrm{snr}}_{\mathrm{av}}}\right)\;, \end{array}$$

(A98) $$\begin{array}{ccc} \Delta_{2}\left({\mathrm{snr}}_{\mathrm{av}}\right){|}_{\beta_{2}>1} & = & \log\left(\frac{{\bar{\mu }}_{1}}{\mu_{2}}\right)+\frac{\left(\mu_{2}-{\bar{\mu }}_{1}\right)\left(\beta_{2}d-1\right)\log e}{\beta_{2}d\left(\beta_{2}-1\right)\tilde{\alpha }\chi }\frac{1}{{\mathrm{snr}}_{\mathrm{av}}}+o\left(\frac{1}{{\mathrm{snr}}_{\mathrm{av}}}\right)\;. \end{array}$$

This yields

(A99) $$\begin{array}{cc} \Delta_{1}\left({\mathrm{snr}}_{\mathrm{av}}\right)+\Delta_{2}\left({\mathrm{snr}}_{\mathrm{av}}\right)\; & =\log\left(\frac{{\bar{\mu }}_{1}{\bar{\mu }}_{2}}{\mu_{1}\mu_{2}}\right)-\left(\frac{\beta_{1}d-1}{\beta_{1}\left(\beta_{1}-1\right)}+\frac{\beta_{2}d-1}{\beta_{2}\left(\beta_{2}-1\right)\tilde{\alpha }}\right)\frac{{\bar{\mu }}_{12}\log e}{d\chi }\frac{1}{{\mathrm{snr}}_{\mathrm{av}}}+o\left(\frac{1}{{\mathrm{snr}}_{\mathrm{av}}}\right)\;, \end{array}$$

hence, for ${\mathrm{snr}}_{\mathrm{av}}\to \infty$,

(A100) $$\begin{array}{cc} 2^{\Delta_{1}\left({\mathrm{snr}}_{\mathrm{av}}\right)+\Delta_{2}\left({\mathrm{snr}}_{\mathrm{av}}\right)}\; & =\frac{{\bar{\mu }}_{1}{\bar{\mu }}_{2}}{\mu_{1}\mu_{2}}\left(1-K_{1}\;{\bar{\mu }}_{12}\;\frac{1}{{\mathrm{snr}}_{\mathrm{av}}}\right)+o\left(\frac{1}{{\mathrm{snr}}_{\mathrm{av}}}\right)\;, \end{array}$$

where we used the approximation $e^{x}\approx 1+x$, x≪1 and

(A101) $$K_{1}≜\left(\frac{\beta_{1}d-1}{\beta_{1}\left(\beta_{1}-1\right)}+\frac{\beta_{2}d-1}{\beta_{2}\left(\beta_{2}-1\right)\tilde{\alpha }}\right)\cdot \frac{1}{d\chi }\;.$$

Next, considering the log(·) term in (A93), it is concluded (following some algebra) that, for ${\mathrm{snr}}_{\mathrm{av}}\to \infty$,

(A102) $$\begin{array}{cc} \log\left(1-\frac{\mu_{1}\mu_{2}}{{\bar{\mu }}_{12}}\left(2^{\Delta_{1}\left({\mathrm{snr}}_{\mathrm{av}}\right)+\Delta_{2}\left({\mathrm{snr}}_{\mathrm{av}}\right)}-1\right)\right)\; & =-\log{\mathrm{snr}}_{\mathrm{av}}+\log\left({\bar{\mu }}_{1}{\bar{\mu }}_{2}K_{1}\right)+o\left(1\right)\;. \end{array}$$

Combining (A102), (A94) and (A93), while recalling that $S_{\infty ,1}=S_{\infty ,2}=1$ for $\beta_{1},\beta_{2}>1$ (cf. (A60)), we obtain, for ${\mathrm{snr}}_{\mathrm{av}}\to \infty$,

(A103) $$\begin{array}{cc} C_{\mathrm{sum}}^{\mathrm{ub}}\left({\mathrm{snr}}_{\mathrm{av}}\right)\; & =2\log{\mathrm{snr}}_{\mathrm{av}}-\left(L_{\infty ,1}^{\mathrm{opt}}-\log\chi \right)-\left(L_{\infty ,2}^{\mathrm{opt}}-\log\left(\tilde{\alpha }\chi \right)\right)-\log\left({\bar{\mu }}_{1}{\bar{\mu }}_{2}\right)\\ \; & -\log{\mathrm{snr}}_{\mathrm{av}}+\log\left({\bar{\mu }}_{1}{\bar{\mu }}_{2}\right)+\log K_{1}+o\left(1\right)\\ \; & =\log{\mathrm{snr}}_{\mathrm{av}}-\left(L_{\infty ,1}-\log\chi \right)-\left(L_{\infty ,2}-\log\left(\tilde{\alpha }\chi \right)\right)+\log K_{1}+o\left(1\right)\;. \end{array}$$

Hence, for $\beta_{1},\beta_{2}>1$, the high-SNR slope of the sum-rate upper bound (A93) (equivalently, (28)) reads

(A104) $$S_{\infty }^{\mathrm{ub}}=1\;,$$

while the high-SNR power offset satisfies

(A105) $$\begin{array}{cc} L_{\infty }^{\mathrm{ub}}\; & =L_{\infty ,1}+L_{\infty ,2}-\log\left(\tilde{\alpha }\chi^{2}K_{1}\right)\;. \end{array}$$

Finally, noting that

(A106) $$\begin{array}{cc} \tilde{\alpha }\chi^{2}K_{1}\; & =\left(\frac{\left(\beta_{1}d-1\right)\tilde{\alpha }}{\beta_{1}\left(\beta_{1}-1\right)}+\frac{\beta_{2}d-1}{\beta_{2}\left(\beta_{2}-1\right)}\right)\cdot \frac{\chi }{d}\;, \end{array}$$

we obtain (64), which completes the proof.

## Appendix G. RS-CDMA: Achievable Region for Two User Classes

In this appendix, we derive the achievable region for RS-CDMA in the large-system limit following the analysis in [41]. The system model for RS-CDMA can still be described via (1), where we now assume that the entries of the signature matrices are i.i.d. random variables with zero mean, unit variance and finite fourth moment. We also set d=N to comply with the setting considered in [41]. Referring to (8) and starting with the individual rate constraints, we have, from ([41], Section IV), that

(A107) $$\begin{array}{ccc} \frac{1}{N}I\left(x_{1};y|x_{2},{\left\{A_{i}\right\}}_{i=1,2}\right) & \underset{N\to \infty }{\to } & C_{\mathrm{RS}}\left(\beta_{1},{\mathrm{snr}}_{1}\right) \end{array}$$

(A108) $$\begin{array}{ccc} \frac{1}{N}I\left(x_{2};y|x_{1},{\left\{A_{i}\right\}}_{i=1,2}\right) & \underset{N\to \infty }{\to } & C_{\mathrm{RS}}\left(\beta_{2},{\mathrm{snr}}_{2}\right)\;, \end{array}$$

where

(A109) CRS(βi,snri)=βilog1+snri−14F(snri,βi)βlog(1+log1+βisnri−14F(snri,βi)−loge4snriF(snri,βi),i=1,2,

and F(·,·) is defined in (6) [40].

To proceed with the analysis of the sum-rate constraint in (8), we introduce the probability mass function (pmf) as

(A110) $$p\left(g\right)=\left\{\begin{array}{cc} \frac{\beta_{1}}{\beta_{1}+\beta_{2}}=\frac{\beta_{1}}{\beta }, & g=\sqrt{\frac{{\mathrm{snr}}_{1}}{{\mathrm{snr}}_{\mathrm{av}}}}\;,\\ \frac{\beta_{2}}{\beta_{1}+\beta_{2}}=\frac{\beta_{2}}{\beta }, & g=\sqrt{\frac{{\mathrm{snr}}_{2}}{{\mathrm{snr}}_{\mathrm{av}}}}\;, \end{array}\right.$$

where, as in (19),

(A111) $${\mathrm{snr}}_{\mathrm{av}}≜\frac{\beta_{1}}{\beta }{\mathrm{snr}}_{1}+\frac{\beta_{2}}{\beta }{\mathrm{snr}}_{2}\;.$$

Then, in the large-system limit, while relying on the strong law of large numbers (SLLN), the received signal in (1) is well represented by

(A112) $$y=\sqrt{\frac{{\mathrm{snr}}_{\mathrm{av}}}{N}}A_{\mathrm{RS}}Gx+z\;,$$

where the N×K matrix $A_{\mathrm{RS}}=\left[A_{1}\;A_{2}\right]$ has i.i.d. zero-mean entries with unit variance and finite fourth moment, $G=\mathrm{diag}\left(g_{1},\cdots ,g_{K}\right)$ is a diagonal random matrix with i.i.d. entries distributed according to (A110), $x∼CN(0,I_{K})$ denotes the input vector comprising both classes of users and $z∼CN(0,I_{N})$ denotes the AWGN. Next, by ([41], Theorem IV.1), we have

(A113) $$\frac{1}{N}I\left(x_{1},x_{2};y|{\left\{A_{i}\right\}}_{i=1,2}\right)\underset{N\to \infty }{\to }C_{\mathrm{RS}}^{\mathrm{sum}}\;,$$

where

(A114) $$C_{\mathrm{RS}}^{\mathrm{sum}}=C_{\mathrm{RS}}^{\mathrm{mmse}}+\log\frac{1}{\eta_{\mathrm{RS}}}+\left(\eta_{\mathrm{RS}}-1\right)\log e\;,$$

(A115) $$C_{\mathrm{RS}}^{\mathrm{mmse}}=\beta_{1}\log\left(1+{\mathrm{snr}}_{1}\eta_{\mathrm{RS}}\right)+\beta_{2}\log\left(1+{\mathrm{snr}}_{2}\eta_{\mathrm{RS}}\right)\;,$$

and $\eta_{\mathrm{RS}}$ is the positive solution to

(A116) $$\eta_{\mathrm{RS}}+\beta_{1}\frac{\eta_{\mathrm{RS}}{\mathrm{snr}}_{1}}{1+\eta_{\mathrm{RS}}{\mathrm{snr}}_{1}}+\beta_{2}\frac{\eta_{\mathrm{RS}}{\mathrm{snr}}_{2}}{1+\eta_{\mathrm{RS}}{\mathrm{snr}}_{2}}=1\;.$$

The explicit dependence of $C_{\mathrm{RS}}^{\mathrm{sum}}$, $C_{\mathrm{RS}}^{\mathrm{mmse}}$ and $\eta_{\mathrm{RS}}$ on ${\left\{\beta_{i},{\mathrm{snr}}_{i}\right\}}_{i=1,2}$ was omitted here for notational simplicity. Note that (A116) boils down to the 3rd order polynomial equation

(A117) $$a_{3}\eta_{\mathrm{RS}}^{3}+a_{2}\eta_{\mathrm{RS}}^{2}+a_{1}\eta_{\mathrm{RS}}+a_{0}=0\;,$$

where

(A118a) $$\begin{array}{ccc} a_{3} & = & {\mathrm{snr}}_{1}{\mathrm{snr}}_{2}\;, \end{array}$$

(A118b) $$\begin{array}{ccc} a_{2} & = & {\mathrm{snr}}_{1}{\mathrm{snr}}_{2}\left(\beta_{1}+\beta_{2}-1\right)+{\mathrm{snr}}_{1}+{\mathrm{snr}}_{2}\;, \end{array}$$

(A118c) $$\begin{array}{ccc} a_{1} & = & \left(\beta_{1}-1\right){\mathrm{snr}}_{1}+\left(\beta_{2}-1\right){\mathrm{snr}}_{2}+1\;, \end{array}$$

(A118d) $$\begin{array}{ccc} a_{0} & = & -1\;. \end{array}$$

Combining (A107), (A108) and (A113), we finally conclude that the achievable rate region for RS-CDMA in the large-system limit reads

(A119) $$\begin{array}{cc} R^{\mathrm{RS}}=\{\left(R_{1},R_{2}\right):R_{1} & \leq C_{\mathrm{RS}}\left(\beta_{1},{\mathrm{snr}}_{1}\right),\\ R_{2} & \leq C_{\mathrm{RS}}\left(\beta_{2},{\mathrm{snr}}_{2}\right),\\ R_{1}+R_{2} & \leq C_{\mathrm{RS}}^{\mathrm{sum}}\}\;. \end{array}$$

## Appendix H. The High-SNR Regime: Some Fundamental Observations

In this appendix, we pursue a somewhat refined high-SNR approximation of $C^{\mathrm{opt}}\left(\mathrm{snr},\beta ,d\right)$, as given by (5) (we omit subscripts in the following since the analysis holds for either i=1 or i=2). The observations made here serve as a fundamental tool for the high-SNR analysis of the sum-rate lower and upper bounds of Propositions 1 and 2 (see Appendices Appendix E and Appendix F, respectively).

### Appendix H.1. β = 1

We start by considering the case where β=1; hence, (cf. Theorem 1) α=γ, $\tilde{\beta }=1$ and $\zeta =\frac{d}{\alpha }$, yielding

(A120) $$C^{\mathrm{opt}}\left(\mathrm{snr},1,d\right)=\frac{d}{2}\log\left(1+2\alpha \mathrm{snr}-\frac{1}{4}F\left(\alpha \mathrm{snr},1\right)\right)-\frac{d-2}{2}\log\left(\frac{{\left(1+d\;\mathrm{snr}\right)}^{2}}{G(\alpha \mathrm{snr},\frac{d}{\alpha },1)}\right)\;.\;$$

Note that

(A121) $$\begin{array}{cc} F(\alpha \mathrm{snr},1)\; & ={\left(\sqrt{4\alpha \mathrm{snr}+1}-1\right)}^{2}=2\alpha \mathrm{snr}\left(2-\frac{\sqrt{4\alpha \mathrm{snr}+1}}{\alpha \mathrm{snr}}+\frac{1}{\alpha \mathrm{snr}}\right)\;, \end{array}$$

while

(A122) $$\begin{array}{cc} G(\alpha \mathrm{snr},\frac{d}{\alpha },1)\; & ={\left(\frac{\sqrt{d\left(4\alpha \mathrm{snr}+1\right)}-\sqrt{d-4\alpha }}{\sqrt{d}-\sqrt{d-4\alpha }}\right)}^{2}=d\left(4\alpha \mathrm{snr}+1\right){\left(\frac{1-\frac{\sqrt{d-4\alpha }}{\sqrt{d\left(4\alpha \mathrm{snr}+1\right)}}}{\sqrt{d}-\sqrt{d-4\alpha }}\right)}^{2}\;. \end{array}$$

Substituting (A121) back into the first logarithmic term in (A120) yields (following some algebra)

(A123) log1+2αsnr−14F(αsnr,1)=logsnr+logα+log1+4αsnr+12αsnr−12αsnr=logsnr+log1+1αsnr1+4αsnr+12αsnr−12αsnr→snr→∞logsnr+log1−1d+dloged−11snr+o(1snr),

where we use the following approximation:

(A124) $$\log\left(1+x\right)=\log x+\log\left(1+\frac{1}{x}\right)\to \log x+\frac{\log e}{x}+o\left(\frac{1}{x}\right)\;,\;x\gg 1\;.$$

Similarly, using (A122), we obtain, for the second logarithmic term in (A120),

(A125) log(1+dsnr)2G(αsnr,dα,1)=2log(1+dsnr)−log(4αsnr+1)−2logd+2=−2log1−d−2d4αsnr+1→snr→∞logsnr−log1−1d+(d−2)loged(d−1)1snr+o(1snr).

Substituting the above high-SNR approximations back into (A120), we finally obtain

(A126) $$\begin{array}{cc} C^{\mathrm{opt}}\left(\mathrm{snr},1,d\right) & \underset{\mathrm{snr}\to \infty }{\to }\log\mathrm{snr}+\left(d-1\right)\log\left(1-\frac{1}{d}\right)+\frac{2\sqrt{d-1}\log e}{\sqrt{d}}\frac{1}{\sqrt{\mathrm{snr}}}+o\left(\frac{1}{\sqrt{\mathrm{snr}}}\right)\;. \end{array}$$

### Appendix H.2. β > 1

For β>1, we investigate $C^{\mathrm{opt}}\left(\mathrm{snr},\beta ,d\right)$ by directly considering its integral form (see [39], ([38], Theorems 2 and 3), (A31) and the corresponding discussion and notation in Appendix C)

(A127) $$C^{\mathrm{opt}}\left(\mathrm{snr},\beta ,d\right)=\int_{0}^{\infty }\log\left(1+\mathrm{snr}\lambda \right)\rho \left(\lambda ,\beta ,d\right)\;d\lambda \;,$$

where

(A128) $$\rho \left(\lambda ,\beta ,d\right)={\left[1-\beta \right]}^{+}\delta \left(\lambda \right)+\frac{\beta d}{2\pi }\frac{\sqrt{{\left[\lambda -\lambda^{-}\right]}^{+}{\left[\lambda^{+}-\lambda \right]}^{+}}}{\lambda (\beta d-\lambda )}\;.$$

Note that, for β>1, the density ρ(λ,β,d) has a *finite strictly positive support* $\left(\lambda^{-},\lambda^{+}\right)$; hence, $C^{\mathrm{opt}}\left(\mathrm{snr},\beta ,d\right)$ can be equivalently expressed as

(A129) $$\begin{array}{cc} C^{\mathrm{opt}}\left(\mathrm{snr},\beta ,d\right) & =\int_{\lambda^{-}}^{\lambda^{+}}\log\left(\mathrm{snr}\lambda \right)\rho \left(\lambda ,\beta ,d\right)\;d\lambda +\int_{\lambda^{-}}^{\lambda^{+}}\log\left(1+\frac{1}{\mathrm{snr}\lambda }\right)\rho \left(\lambda ,\beta ,d\right)\;d\lambda \\ \; & =\log\mathrm{snr}+\int_{\lambda^{-}}^{\lambda^{+}}\log\left(\lambda \right)\rho \left(\lambda ,\beta ,d\right)\;d\lambda +\int_{\lambda^{-}}^{\lambda^{+}}\log\left(1+\frac{1}{\mathrm{snr}\lambda }\right)\rho \left(\lambda ,\beta ,d\right)\;d\lambda \;. \end{array}$$

Therefore, we approximate $C^{\mathrm{opt}}\left(\mathrm{snr},\beta ,d\right)$ in the high-SNR regime as

(A130) $$C^{\mathrm{opt}}\left(\mathrm{snr},\beta ,d\right)=\log\mathrm{snr}+\int_{\lambda^{-}}^{\lambda^{+}}\log\left(\lambda \right)\rho \left(\lambda ,\beta ,d\right)\;d\lambda +\frac{\log e}{\mathrm{snr}}\int_{\lambda^{-}}^{\lambda^{+}}\frac{1}{\lambda }\;\rho \left(\lambda ,\beta ,d\right)\;d\lambda +o\left(\frac{1}{\mathrm{snr}}\right)\;.$$

The two integrals in (A130) can be solved in closed form and read

(A131) $$\begin{array}{cc} \int_{\lambda^{-}}^{\lambda^{+}}\log\left(\lambda \right)\rho \left(\lambda ,\beta ,d\right)\;d\lambda \; & =-\left(\beta -1\right)\log\left(\beta -1\right)+\beta \log\beta +\left(\beta d-1\right)\log\left(1-\frac{1}{\beta d}\right)\;, \end{array}$$

(A132) $$\int_{\lambda^{-}}^{\lambda^{+}}\frac{1}{\lambda }\;\rho \left(\lambda ,\beta ,d\right)\;d\lambda =\frac{\beta d-1}{\beta d(\beta -1)}\;.$$

Hence, we conclude that

(A133) Copt(snr,β,d)→snr→∞logsnr−(β−1)log(β−1)+βlogβ+(βd−1)log1−1βdCopt(snr,1,d)→snr→∞+(βd−1)logeβd(β−1)1snr+o(1snr),β>1.

### Appendix H.3. 2 d≤ β < 1

Using the subscript ${\left(\cdot \right)}_{u}$ to designate quantities corresponding to an underloaded setting (β<1), we start by noting that, in the underloaded regime, $C_{u}^{\mathrm{opt}}\left(\mathrm{snr},\beta ,d\right)$ can be shown to satisfy the relation

(A134) $$C_{u}^{\mathrm{opt}}\left(\mathrm{snr},\beta ,d\right)=\beta C^{\mathrm{opt}}\left(\beta \mathrm{snr},\beta_{\mathrm{ol}},d_{\mathrm{ol}}\right)\;,$$

where we additionally define $\beta_{\mathrm{ol}}≜\frac{1}{\beta }$ and $d_{\mathrm{ol}}≜\beta d$. Here, $C^{\mathrm{opt}}\left(\beta \mathrm{snr},\beta_{\mathrm{ol}},d_{\mathrm{ol}}\right)$ specifies the achievable throughput in an *overloaded* single-class setting with the corresponding parameters (see the discussion in Section 3.1). Hence, $C_{u}^{\mathrm{opt}}\left(\mathrm{snr},\beta ,d\right)$ can be expressed in an integral form as

(A135) $$C_{u}^{\mathrm{opt}}\left(\mathrm{snr},\beta ,d\right)=\beta \int_{0}^{\infty }\log\left(1+\beta \mathrm{snr}\lambda_{\mathrm{ol}}\right)\rho \left(\lambda_{\mathrm{ol}},\beta_{\mathrm{ol}},d_{\mathrm{ol}}\right)\;d\lambda_{\mathrm{ol}}\;,$$

where $\rho (\lambda_{\mathrm{ol}},\beta_{\mathrm{ol}},d_{\mathrm{ol}})$ is as in (A128) with the obvious change in parameters. Since $\beta_{\mathrm{ol}}>1$, the density $\rho (\lambda_{\mathrm{ol}},\beta_{\mathrm{ol}},d_{\mathrm{ol}})$ has a finite strictly positive support and $C_{u}^{\mathrm{opt}}\left(\mathrm{snr},\beta ,d\right)$ can be equivalently rewritten as

(A136) Cuopt(snr,β,d)=β∫λol−λol+log(snrβλol)ρ(λol,βol,dol)dλol=+β∫λol−λol+log(1+1snrβλol)ρ(λol,βol,dol)dλol=βlogsnr+β∫λol−λol+log(βλol)ρ(λol,βol,dol)dλollogsnr+β∫λol−λol+log(1+1snrβλol)ρ(λol,βol,dol)dλol.

Hence, we approximate $C_{u}^{\mathrm{opt}}\left(\mathrm{snr},\beta ,d\right)$ in the high-SNR regime as

(A137) Cuopt(snr,β,d)=βlogsnr+β∫λol−λol+log(βλol)ρ(λol,βol,dol)dλollogsnr+βlogesnr∫λol−λol+1βλolρ(λol,βol,dol)dλol+o(1snr).

The two integrals in (A137) can be solved in closed form, yielding

(A138) $$\int_{\lambda_{\mathrm{ol}}^{-}}^{\lambda_{\mathrm{ol}}^{+}}\log\left(\beta \lambda_{\mathrm{ol}}\right)\rho \left(\lambda_{\mathrm{ol}},\beta_{\mathrm{ol}},d_{\mathrm{ol}}\right)\;d\lambda_{\mathrm{ol}}=\left(d-1\right)\log\left(1-\frac{1}{d}\right)-\left(\frac{1}{\beta }-1\right)\log\left(1-\beta \right)\;,$$

(A139) $$\int_{\lambda_{\mathrm{ol}}^{-}}^{\lambda_{\mathrm{ol}}^{+}}\frac{1}{\beta_{\mathrm{ol}}\lambda_{\mathrm{ol}}}\;\rho \left(\lambda_{\mathrm{ol}},\beta_{\mathrm{ol}},d_{\mathrm{ol}}\right)\;d\lambda =\frac{\beta^{2}\left(d-1\right)}{d(1-\beta )}\;,$$

and the high-SNR approximation for $C_{u}^{\mathrm{opt}}\left(\mathrm{snr},\beta ,d\right)$ thus reads

(A140) Cuopt(snr,β,d)→snr→∞βlogsnr+(d−1)log21−1d−1β−1log(1−β)logsnr+β3(d−1)loged(1−β)1snr+o1snr.
